# Design, content, and fieldwork procedures of the COVID‐19 Psychological Research Consortium (C19PRC) Study – Wave 4

**DOI:** 10.1002/mpr.1899

**Published:** 2021-11-05

**Authors:** Orla McBride, Sarah Butter, Jamie Murphy, Mark Shevlin, Todd K. Hartman, Kate M. Bennett, Thomas V. A. Stocks, Alex Lloyd, Ryan McKay, Jilly Gibson‐Miller, Liat Levita, Liam Mason, Anton P. Martinez, Philip Hyland, Frédérique Vallières, Thanos Karatzias, Carmen Valiente, Carmelo Vazquez, Richard P. Bentall

**Affiliations:** ^1^ School of Psychology Ulster University Coleraine Northern Ireland; ^2^ University of Sheffield Sheffield England; ^3^ University of Manchester Manchester England; ^4^ University of Liverpool Liverpool England; ^5^ Royal Holloway University of London London England; ^6^ University College London London England; ^7^ Maynooth University Maynooth Republic of Ireland; ^8^ Trinity College Dublin Dublin Republic of Ireland; ^9^ Napier University Edinburgh Scotland; ^10^ Complutense University of Madrid Madrid Spain

**Keywords:** COVID‐19, general population, longitudinal, psychological, survey methodology

## Abstract

**Objectives:**

This paper outlines fieldwork procedures for Wave 4 of the COVID‐19 Psychological Research Consortium (C19PRC) Study in the UK during November–December 2020.

**Methods:**

Respondents provided data on socio‐political attitudes, beliefs, and behaviours, and mental health disorders (anxiety, depression, and posttraumatic stress). In Phase 1, adults (*N* = 2878) were reinvited to participate. At Phase 2, new recruitment: (i) replenished the longitudinal strand to account for attrition; and (ii) oversampled from the devolved UK nations to facilitate robust between‐country analyses for core study outcomes. Weights were calculated using a survey raking algorithm to ensure the longitudinal panel was representative of the baseline sample characteristics.

**Results:**

In Phase 1, 1796 adults were successfully recontacted and provided full interviews at Wave 4 (62.4% retention rate). In Phase 2, 292 new respondents were recruited to replenish the panel, as well as 1779 adults from Wales, Scotland, and Northern Ireland, who were representative of the socio‐political composition of the adult populations in these nations. The raking procedure successfully re‐balanced the longitudinal panel to within 1% of population estimates for selected socio‐demographic characteristics.

**Conclusion:**

The C19PRC Study offers a unique opportunity to facilitate and stimulate interdisciplinary research addressing important public health questions relating to the COVID‐19 pandemic.

## 1 INTRODUCTION

The ‘first wave’ of the COVID‐19 pandemic in the UK was abating by the summer of 2020 (Kontis et al., 2020), and citizens were experiencing respite from the government‐imposed restrictions on social, economic, and educational related activities that had been in place since March 2020 to control the spread of the virus. For example, in August 2020, ‘shielding’ initiatives requiring ∼2 million elderly and/or medically vulnerable individuals to self‐isolate to avoid contracting COVID‐19 were paused across most of the UK (UK Government, 2020a). A month‐long ‘*Eat Out to Help Out*’ scheme offering discounted meals at indoor venues was launched to support the reopening of businesses (UK Government, 2020c). Employers were actively encouraged to reassure employees that it was safe to return to office workplaces (UK Government, 2020e). Also, the majority of primary and secondary schools re‐opened for face‐to‐face teaching for all students (UK Government, 2020b).

**FIGURE 1:**
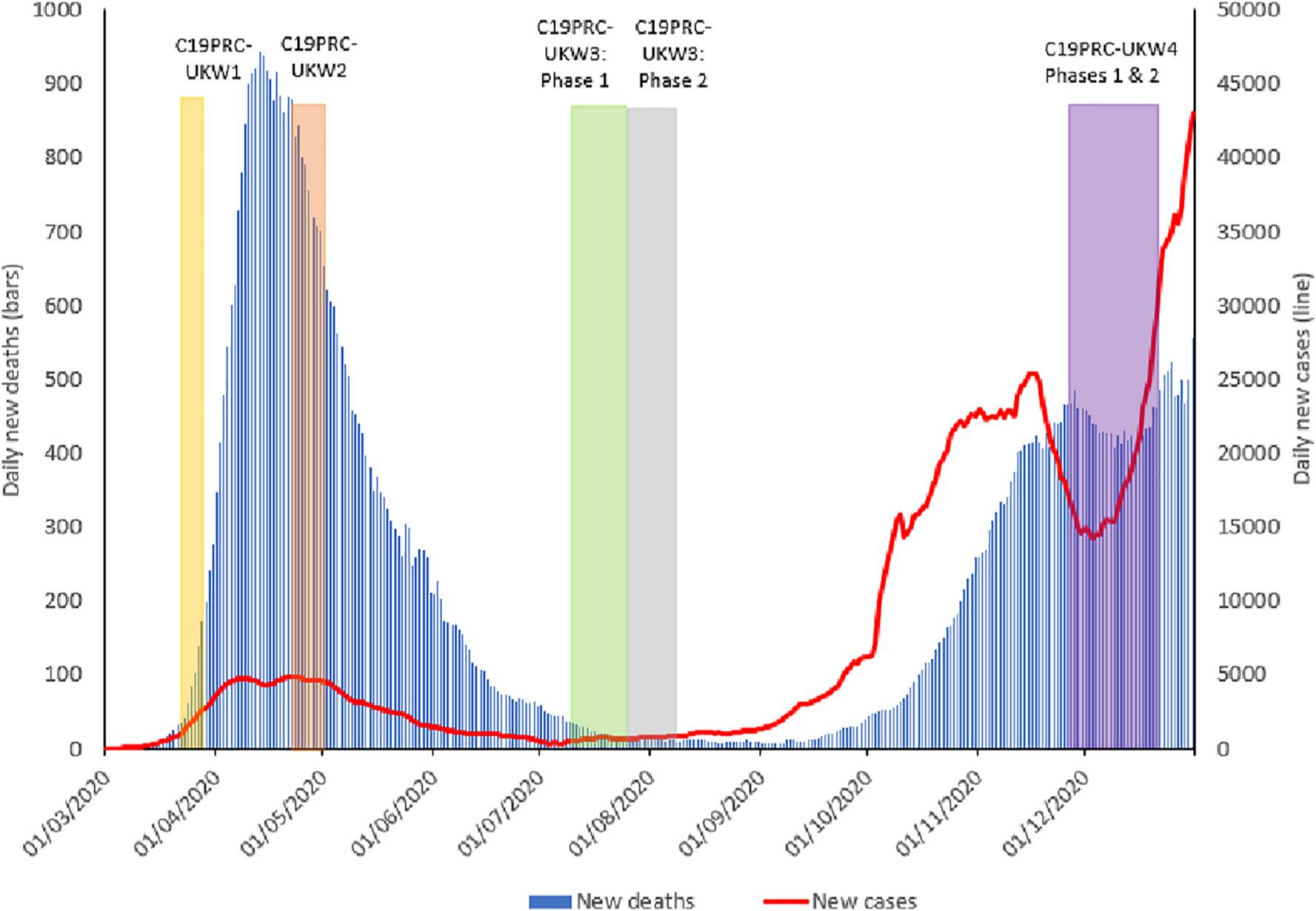
Graphical presentation of the number of daily COVID‐19 cases and deaths in the UK, sourced from Our World in Data, 2020, aligned to the COVID‐19 Psychological Research Consortium (C19PRC) Study survey waves. *Note*: New daily deaths and cases depicted as 7‐day rolling average

During this time, concerns were raised by public health experts about the potential impact of an equally, if not more, devastating ‘second wave’, evidence of which was already being reported in parts of Europe, which was predicted to hit the UK by autumn 2020 (Academy of Medical Sciences, 2020; Looi, 2020; Mahase, 2020; Middleton et al., 2020). By October 2020, as the UK COVID‐19 reproduction rate was estimated to be between 1.3 and 1.5, signalling high levels of infection transmissibility in communities, one‐quarter of the UK population (∼16.8 million citizens) was forced back into lockdown (UK Government, 2020d). The Office for National Statistics (2020) reported that COVID‐19 cases were increasing rapidly, and that COVID‐19 related hospital admissions were close to the peak of the ‘first wave’ in spring 2020 (see Figure 1). On 5 November 2020, the UK Prime Minister announced a second national lockdown for England, initially for 4 weeks (UK Government, 2020g), and the government ‘furlough scheme’, which provides up to 80% income support for unemployed workers, was extended until March 2021 (UK Government, 2020f). On 2 December 2020, the lockdown was replaced with a revised regional COVID‐19 tier‐system, which re‐imposed regulations on social gatherings (UK Government, 2020h). On this same date, the UK became the first country in the world to approve the Pfizer‐BioNTech vaccine (Ledford et al., 2020) and the vaccination rollout commenced 6 days later. The Oxford‐AstraZeneca vaccine was anticipated to be approved and subsequently deployed in January 2021 (UK Government Coronavirus (COVID‐19) in the UK, 2021).

In late 2020, whilst grappling with preparations for an upcoming COVID‐19 surge, as well as planning the population‐wide COVID‐19 vaccination rollout, the UK government was facing a major historical political event: the end of the Brexit transition period on 31 December 2020 (Wright & Etherington, 2020). The crippling economic impacts of the protracted pandemic, compounded by uncertainty surrounding the short‐to‐medium term impacts of Brexit, was predicted to widen existing regional inequalities in the UK (Bhattacharjee et al., 2020; Petrie & Norman, 2020). The decision to hold the 2016 European Union referendum, and the voting outcome of that referendum, had already resulted in societal division across the UK and Europe (Hobolt, 2018; Outhwaite, 2017), particularly in relation to views on culture, migration, racism, national identity, and political ideologies (Bachmann & Sidaway, 2016; Corbett, 2016). Moreover, the pandemic itself, coupled with states' initiatives to control the spread of the disease across populations, may have contributed substantially to the rise of nationalism and its social relevance on a global scale (Bieber, 2020; Woods et al., 2020).

It is against this backdrop that the fourth wave of the C19PRC Study (hereafter referred to as C19PRC‐UKW4) was conducted in the UK during November–December 2020. Established in March 2020, the C19PRC Study is a dynamic, online, longitudinal, multi‐country study which aims to evaluate the psychological, socio‐economic, and political impacts of the pandemic on the lives of adults living in the UK, Republic of Ireland, Spain, and Italy (McBride et al., 2020). The UK‐strand of the C19PRC Study (C19PRC‐UK) is the ‘parent’ strand of the Consortium upon which other countries model their survey design, content, and fieldwork procedures. Methodological reports for the C19PRC Study in the UK (Wave 1, March 2020; Wave 2, April 2020; Wave 3, July/August 2020) and other countries are available elsewhere (Bruno et al., 2021; Hyland, Vallières, Shevlin, et al., 2021; McBride et al., 2020, 2021; Valiente et al., 2020, 2021).

### 1.1 Brief overview of C19PRC Study methodological framework for the C19PRC‐UKW4

Collecting data on adults' mental health difficulties (e.g. anxiety, depression, and traumatic stress) at each survey wave, using standardised and validated measures, is a fundamental design feature of the C19PRC Study. Our study has spearheaded COVID‐19‐related mental health research through the production of timely, high‐quality research articles, the findings of which have already demonstrated relatively stable prevalence estimates for depression, anxiety, and COVID‐19 traumatic stress during the first 6 months of the pandemic in the UK and Ireland (Hyland, Vallières, Daly, et al., 2021; Shevlin et al., 2021).

The C19PRC Study was designed to go beyond the collection of self‐reported survey data at each wave. For example, in this fourth wave, a ‘survey list experiment’ was conducted to assess respondents' compliance with government‐imposed public health regulations (for details, see Supporting Information S2: Section 2.2.12.2).

Considerable efforts have been made to maximise participant retention at each wave post‐baseline to minimise bias in survey estimates, and this strategy has been successful to date (i.e. ∼60% retention of participants over three waves) (McBride et al., 2021). Moreover, new participants were recruited into the cohort at Wave 3 (C19PRC‐UKW3) so that the cross‐sectional sample at each wave would continue to be (1) representative of the UK adult population (i.e. new participants were recruited to ‘top‐up’ baseline quotas, determined by age, gender, and household income, due to modest levels of attrition) and (2) large enough (*n* > 2K adults) to conduct sub‐group analyses for core study outcomes.

This paper describes the fieldwork procedures for C19PRC‐UKW4. Two key decisions were made during the planning phase: (1) to prioritise collection of data on respondents' socio‐political views, attitudes, and behaviours to assess the combined impact of Brexit and the COVID‐19 pandemic on adults' national identity, and how this might in turn shape their responses to, and experiences of, the pandemic; and (2) to recruit new respondents into the study by oversampling adults from the devolved UK nations (Wales, Scotland, and Northern Ireland), which would facilitate robust between‐country comparisons for a range of important socio‐political outcomes, in addition to the core mental health outcomes. Here, we (i) examine patterns of attrition in the C19PRC Study by this fourth wave and whether these could be predicted by baseline mental‐health attributes, psychological characteristics, as well as socio‐demographic factors; (ii) conduct and assess weighting procedures to manage attrition in the longitudinal panel; (iii) determine the success of sample replenishment and oversampling procedures conducted at C19PRC‐UKW4; and (iv) describe the prevalence of common mental disorders among participants in the C19PRC‐UKW4 sample, as well as their socio‐demographic characteristics and political‐related beliefs and behaviours.

## 2 METHOD

### 2.1 C19PRC‐UKW4: fieldwork procedures

#### 2.1.1 Fieldwork organisation overview

The survey company Qualtrics conducted the fieldwork for C19PRC‐UKW4. Qualtrics partners with over 20 online sample providers to supply a network of diverse, quality respondents to their worldwide client base and, to date, has completed more than 15,000 projects across 2500 universities worldwide.

#### 2.1.2 Procedure

C19PRC‐UKW4 commenced on 25 November 2020, approximately 4 months after the completion of C19PRC‐UKW3 (conducted during July–August 2020). C19PRC‐UKW4 comprised two phases.

Phase 1 comprised two strands. Qualtrics re‐contacted all adults who participated in any previous wave(s) (*N* = 2878) via email, SMS, or in‐app notifications and invited them to participate further in this survey (invitations were tailored to remind adults of their participation in previous survey waves). Only 2025 of these eligible respondents participated at baseline and were being invited to participate in a fourth survey; the remaining 853 first entered the panel at C19PRC‐UKW3 and were being invited to participate in their first follow‐up survey. Phase 1 fieldwork was completed on 22 December 2020.

Fieldwork for Phase 2 (sample replenishment and oversampling) was conducted between 25 November and 19 December 2020. Similar to the process of recruitment at baseline, new participants for Phase 2 were sampled from Qualtrics' partners' existing survey panels and were alerted to the C19PRC‐UKW4 by Qualtrics in one of two ways: (1) they opted to enter studies they were eligible for by signing up to a panel platform; or (2) they received automatic notification through a partner router which alerted/directed them to studies for which they were eligible. To avoid self‐selection bias, survey invitations provided only general information and did not include specific details about the contents of the survey. Participants were required to be adults, able to read and write in English, and resident in the UK. No other exclusion criteria were applied. All panel members routinely received an incentive for survey participation (e.g. gift cards), based on the length of the survey, their specific panellist profile, and target acquisition difficulty, amongst other factors. Qualtrics' partners released invitations in batches and, after the initial invitation was received, respondents who had not completed the survey were sent two reminders to encourage them to participate. The first reminder was sent approximately 36–48 h after the initial survey invite, with the second reminder sent another 36–48 h after this first reminder.

#### 2.1.3 Informed consent process

Participants were informed about the purpose of the C19PRC Study, that their data would be treated in confidence, that geolocating would be used to determine the area in which they lived (in conjunction with their residential postcode stem), and of their right to terminate participation at any time. Participants were also informed that some topics might be sensitive or distressing. Information about how their data would be stored and analysed by the research team was also provided. Participants were also informed that they would be re‐contacted at a later date to invite them to participate in subsequent survey waves. Participants provided informed electronic consent prior to completing the survey and were directed to contact the NHS website upon completion if they had any concerns about COVID‐19.

#### 2.1.4 Compliance with General Data Protection Regulation (GDPR)

C19PRC data will be stored confidentially in line with GDPR. When the study data is deposited with the UK Data Service, location data will be removed and replaced with relevant socioeconomic summary data (e.g. area‐level deprivation and population density data). All other personal data will also be removed.

#### 2.1.5 Quality control

Qualtrics are committed to delivering high‐quality survey data from online survey panels and multiple validation checks are conducted on the C19PRC‐UK data to ensure this target is met. First, the survey is piloted (‘soft launch’; *n* = 100) prior to the fieldwork going live (‘full launch’) to rectify sequencing/coding errors and omissions prior to the full launch. The soft launch also calculate the median survey completion time, which provides an opportunity to tailor the content to ensure the median survey time does not exceed 30 min; this is important to minimise respondent burden and maximise participation over time. For C19PRC‐UKW4, a soft launch was conducted (comprising ∼50 respondents) for each phase. The median survey completion times were 23 min 17 s for Phase 1 and 23 min 7 s for Phase 2. These respondents were excluded from the final sample for that Phase. Given the median times were under the 30‐min threshold, additional measures were included in the survey prior to the full launch. Second, each participant must achieve ‘legitimate respondent status’ upon entry into the survey. This means that the respondent must spend a minimum amount of time completing the survey (i.e. half the median soft launch completion time for that wave) the first time they participate. Respondents who do not achieve this status are flagged as ‘speeders’ and removed from the study. And third, any respondent who does not meet the inclusion criteria, or who does not complete the survey in full, is removed from the final sample for that Phase.

### 2.2 Measures

Table 1 provides an overview of the C19PRC‐UKW4 survey content by Phase (see Supporting Information S2 for details of all measures administered).

**TABLE 1:** Overview of content of C19PRC Study Wave 4 (Phases 1 and 2), United Kingdom (UK), November – December, 2020

Theme	Content	C19PRC Wave 4	
		Phase 1	Phase 2
Demographics	Age, gender, ethnicity, marital status, economic activity, country of residence, country of birth, born in the UK, key/essential worker status, urbanicity^a^, level of education^†^, religion^†^, self‐reported social class, perceived social rank	X	X^†only^
Housing characteristics	Living alone	X	X
	Number of adults living in household	X	X
	Parental and children in the home status	X	X
	Housing tenure^a^	X	X
	Residential details (type of property; number of bedrooms; length at property)^a^	X	X
Household finances	Estimated annual gross household income	X	X
	Change in monthly household income during pandemic	X	X
	Use of savings/increasing debt during pandemic	X	X
	Made saving due to pandemic	X	X
	Concern over household finances being negatively affected due to pandemic	X	X
	Perceived future financial security	X	X
	Receiving benefits	X	X
	Difficulty paying bills	X	X
	Food insecurity: pre‐pandemic and currently	X	X
Working hours	Number of hours worked weekly pre/post pandemic	X	X
	Number of hours would like to be working	X	X
Health conditions	Existence of any major underlying health conditions – self		X
	Existence of any major underlying health conditions – immediate family member		X
	Currently pregnant – self (partner)	X	X
	Number of weeks pregnant, if applicable	X	X
	Currently pregnant – immediate family member	X	X
	Self‐rated health	X	X
COVID‐19	Sourcing of information (newspapers, TV, radio, social media, internet, etc.)		X
	Level of trust in information source		X
	Anxiety‐level relating to COVID‐19	X	X
	Confidence in response to COVID‐19 pandemic	X	X
	Perceived threat of COVID‐19	X	X
	Perceived individual risk contracting COVID‐19 over next 6 months	X	X
	Perceived severity of COVID‐19 symptoms if infected/reinfected	X	X
	Experiences of self‐isolation	X	X
	Experiences of children in the home self‐isolating	X	X
	Experience of being infected with COVID‐19 (self and family member/friend)	X	X
	Experience of being tested for COVID‐19 (symptoms/location of testing/diagnosis)	X	
	Knowing someone close (family member/friend) who has tested positive for COVID‐19	X	X
	Knowing someone close (family member/friend) who has tested died due to COVID‐19	X	X
	Behaviour – engagement with social distancing	X	X
	COVID‐19 vaccine acceptability (self)	X	X
	COVID‐19 vaccine acceptability (child)	X	X
	Beliefs about vaccines made available to the public	X	X
	Support/opposition for mandatory vaccination	X	X
	Predicted course of the pandemic	X	X
	Support/opposition for restrictions in case of second wave	X	X
	Perceived compliance with lockdown rule by different demographic groups (e.g. students, migrants, etc.) and nationally	X	X
	Understanding of COVID‐19 restrictions and regulations	X	X
	Perceived importance of factors affecting lockdown decisions	X	X
	Survey list experiment relating to social distancing/adherence to lockdown rules	X	X
Mental Health	Depression: *Patient Health Questionnaire‐9* (Kroenke et al., 2001)	X	X
	Anxiety: *Generalised Anxiety Disorder Scale‐7* (Spitzer et al., 2006)	X	X
	Traumatic Stress *International Trauma Questionnaire* (Cloitre et al., 2018)	X	X
	Paranoia: *Persecution and Deservedness Scale* (Melo et al., 2009)	X	X
	Self‐harm, suicidal thoughts and suicide attempts	X	X
	Treatment for mental health difficulties	X	X
Psychological factors	Personality: *Big‐Five Inventory‐10* (Rammstedt & John, 2007)	X	X
	Loneliness: *Loneliness Scale* (Hughes et al., 2004)	X	X
	Religiosity: *Monotheist and Atheist Beliefs Scale* (Alsuhibani et al., 2021)	X	X
	Empathy: *Interpersonal Reactivity Index* (Davis, 1980)		X
	Conspiracy theories: *Conspiracy Mentality Scale* (Imhoff & Bruder, 2014)	X	X
	Hopefulness: *Brief‐H‐Positive* (Fraser et al., 2014)	X	X
	Happiness: degree of happiness yesterday	X	X
	Life satisfaction	X	X
	Aspects of life better/worse since pandemic	X	X
	Wellbeing: *Warwick‐Edinburgh Mental Wellbeing Scale (WEMWBS, short 7‐item version)* (Stewart‐Brown et al., 2009)	X	X
	Cognitive reflection task		X
	Social engagement with family and friends (Seeman et al., 2011)	X	
Socio‐political views/related behaviours	Voting behaviour last general Election		X
	Voting behaviour European Referendum		X
	Measure of ‘left‐wing’ or ‘right‐wing’ on social and economic issues		X
	Satisfaction with how government/institutions handling pandemic	X	X
	Patriotism/nationalism	X	X
	Social dominance*: Social Dominance Scale* (Ho et al., 2015)	X	X
	Authoritarianism: *Very Short Authoritarianism Scale* (Bizumic & Duckitt, 2018)	X	X
	Attitudes towards migrants	X	X
	Political party identification		X
	Citizenship	X	X
	National identity (degree of British & Irish identity, importance of identity, pride in identity)	X	X
	National belonginess	X	X
	Positive/Negative feelings towards flags (British, English, Welsh, Scottish, Irish, EU)	X	X
	Positive/Negative feelings towards people of UK and Ireland	X	X
	Place resentment	X	X
	Attitudes towards an Irish border poll (support and predicted voting)	X	X
	Support for an all‐Ireland COVID‐19 strategy	X	X
	Attitudes towards a Scottish Independence Referendum (support and predicted voting)	X	X
	English identity (Northerner/Southerner)	X	X
	North/South England resentment	X	X
	UK remaining united – perceived likelihood and preference	X	X
	Languages (Scots, Ulster Scots, Scot Gaelic, Welsh & Irish)	X	X
	Hindsight attitudes towards Brexit	X	X
	Perceived impact of Brexit on UK	X	X
	Brexit predictions	X	X
	EU Referendum voter identification	X	X
	Populism	X	X
Trust	Institutions	X	X
Purchasing behaviours	Increased purchasing for specific items (e.g. dried food) during pandemic	X	X
	Perceptions of supermarket stock levels	X	X
	Purchasing of specific food types (e.g. healthy, convenient, etc.)	X	X
Seasonal items	Food and present affordability at Christmas	X	X
	Food and present availability at Christmas	X	X
	Worry about visiting family/friends over Christmas	X	X
	Perceived difficulty visiting family/friends over Christmas	X	X

*Note*: Refer to Supporting Information S2 for detailed information on all study measures.

^a^ These items only asked at Phase 1 if respondent reported that they had moved home since last completing the survey.

^†^ Level of education and religion were only measured at Phase 2 only.

#### 2.2.1 Study variables

These baseline (C19PRC‐UKW1) variables were used for attrition analyses for C19PRC‐UKW4: gender (females vs. males); age (18–24 years olds vs. 25–34 years, 35–44 years, 45–54 years, 55–64 years, and 65+ years groups); household income (≤£15,490 per annum vs. £15,491–£25,340, £25,341–£38,740, £38,741–£57,903, and ≥£57,931 bands); economic activity (employed vs. other); ethnicity (White vs. other); born in UK (yes vs. no); urbanicity (living in city vs. suburb, town or rural location); education (post‐secondary education vs. other); religion (atheist or agnostic vs. any religion); household composition (living alone vs. other; children <18 years living in household vs. other); physical health (self‐reported chronic health condition vs. other); probable depression diagnosis (score of ≥10 on the *Patient Health Questionnaire‐9* vs. other); probable generalised anxiety diagnosis (score of ≥10 on the *Generalised Anxiety Disorder‐7* vs. other); probable PTSD diagnosis (using the *International Trauma Questionnaire's* diagnostic algorithm for PTSD caseness relating to experience of COVID‐19 vs. other); mental health treatment (current or past treatment for mental health problems vs. other); loneliness (score of ≥6 on the *Loneliness Scal*e); neuroticism (total score on the neuroticism subscale of the *Big‐Five Inventory‐10*); somatisation (total score on the *Patient Health Questionnaire‐15*); resilience (total score on the *Brief Resilience Scale*); paranoia (total score on the *Persecution and Deservedness Scale*); death anxiety (total score on the *Death Anxiety Inventory*); intolerance of uncertainty (total score on the *Intolerance of Uncertainty Scale*); and COVID‐19 anxiety (total score on single item indicator).

In addition, these variables (same categorisation as above) were used to describe the C19PRC‐UKW4 sample characteristics and for attrition analyses for C19PRC‐UKW4: gender; age; household income; ethnicity; economic activity; birthplace; household composition; urbanicity; depression; anxiety; PTSD; physical health condition; and voting opinions and behaviours (i.e. 2016 EU Referendum; Brexit hindsight; UK General Election 2019 vote; and political party affiliation).

### 2.3 Ethical approval

Ethical approval for the project was provided by the University of Sheffield (Reference number 033759).

### 2.4 Data analysis plan and weighting procedures

Four sets of analyses are presented. First, the longitudinal panels starting at (i) baseline, and (ii) the previous wave (C19PRC‐UKW3), were considered separately, and overall, for the purposes of calculating re‐contact rates and conducting attrition analyses. Specifically, a multinomial logistic regression tested the associations between a range of baseline socio‐demographic, mental health conditions, and psychological factors and levels of participation across the four waves of the C19PRC Study (i.e. comparing completion of the baseline survey only to completion in any 2, 3 or all four subsequent waves). For all adults eligible for follow‐up at C19PRC‐UKW4, responders and non‐responders at were compared on a range of socio‐demographic characteristics, using chi‐square tests.

Second, post‐stratification survey weighting was conducted using a technique known as survey raking or sample‐balancing, using the ‘anesrake’ package in R (Pasek & Pasek, 2018). Raking is one common method of adjusting survey data to ensure that the distribution of the characteristics of a given sample closely mirror the known population distribution. In practice, this means the C19PRC‐UKW1 sampling quotas for age, gender, and household income, as well as the baseline proportions achieved for ethnicity, urbanicity, household composition, and being born or raised in the UK, were imposed on the sample of responders obtained at Phase 1, and the raking algorithm was conducted to produce, and iteratively adjust, a weight value for each case in the sample until the sample distribution aligned with the population distribution for the chosen characteristics (DeBell & Krosnick, 2009).

Third, the outcome of recruitment at Phase 2 for the replenishment or ‘top‐up’ strand was assessed by comparing the characteristics of adults in the combined Phase 1 and Phase 2 samples (excluding the oversample) with respect to gender, age, and household income, compared to the target sampling quotas specified at baseline to obtain a nationally representative sample of UK adults. The percentage differences between the baseline and C19PRC‐UKW4 quota bands for gender, age, and household income were calculated.

And fourth, the socio‐demographic, mental health, and political characteristics of the C19PRC‐UKW4 sample were assessed using counts and frequencies (weighted, where appropriate) and comparisons across the sample strands using chi‐square tests.

## 3 RESULTS

**FIGURE 2:**
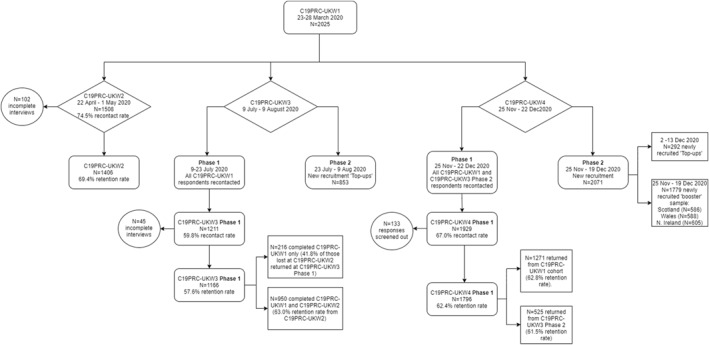
Flowchart of participation in the COVID‐19 Psychological Research Consortium Study (C19PRC) Study, Waves 1–4

Figure 2 illustrates the outcome of recruitment of C19PRC‐UKW4, Phase 1 and Phase 2.

### 3.1 Outcome of Phase 1 recruitment and attrition analyses

The median survey completion time for Phase 1 was 28 min 42 s. Of the 2025 adults who participated at baseline, 1271 (62.8% recontact rate) were successfully recontacted at this fourth wave. Only 15% (*N* = 304) of the baseline sample had not participated in any subsequent wave; 38.1% (*N* = 771) of baseline respondents had participated in all three subsequent follow‐up waves, with 580 respondents (28.6% of baseline respondents) and 370 (18.3%) participants in any one or two follow‐up surveys, respectively.

As presented in Table 2, compared to respondents who were lost to follow‐up after baseline, respondents who participated in all four survey waves were characterised by being older in age (i.e. lower odds of being in age bands 18–54 years), male (OR = 1.43, 95% CI 1.02–2.00), not being in the lowest household income bracket (OR = 0.54, 95% CI 0.30–0.98), having dependent children living in the household (OR = 1.75, 95% CI 1.20–2.56), experiencing lower levels of somatisation (OR = 0.93; 95% CI 0.90–0.96) and paranoia (OR = 0.95; 95% CI 0.92–0.99), but higher levels of intolerance of uncertainty (OR = 1.04; 95% CI = 1.01–1.06). No other baseline characteristics uniquely predicted participation in only one or two survey waves post‐baseline (see Table 2).

**TABLE 2:** Respondent characteristics at baseline (C19PRC‐UKW1, March 2020) predicting participation in all four waves of the C19PRC Study across April–December 2020 (*N* = 2025)

C19PRC‐UKW1 characteristics	Any 2 waves (*n* = 370)	Any 3 waves (*n* = 580)	All 4 waves (*n* = 771)
	OR (95% CI)		
Gender^a^			
Male	1.25 (0.88–1.78)	1.10 (0.79–1.54)	1.43 (1.02–2.00)*
Female	‐	‐	‐
Age group (years)			
18–24	0.15 (0.06–0.38)***	0.05 (0.02–0.13)***	0.01 (0.01–0.03)***
25–34	0.30 (0.12–0.75)**	0.19 (0.08–0.44)***	0.07 (0.03–0.15)***
35–44	0.45 (0.18–1.13)	0.26 (0.11–0.61)**	0.15 (0.06–0.34)***
45–54	0.69 (0.27–1.75)	0.49 (0.21–1.15)	0.36 (0.16–0.83)*
55–64	0.96 (0.34–2.67)	0.95 (0.37–2.45)	0.90 (0.36–2.26)
65+	‐	‐	‐
2019 household income			
≤£15.490	0.58 (0.32–1.06)	0.59 (0.33–1.06)	0.54 (0.30–0.98)*
£15,491–£25,340	0.57 (0.32–0.99)*	0.65 (0.38–1.10)	0.67 (0.39–1.15)
£25,341–£38,740	0.59 (0.34–1.02)	0.65 (0.39–1.11)	0.70 (0.41–1.19)
£38,741–£57,903	0.89 (0.51–1.54)	0.72 (0.42–1.23)	1.02 (0.60–1.73)
≥£57,931	‐	‐	‐
Employment			
Employed	1.19 (0.80–1.78)	1.15 (0.79–1.68)	1.09 (0.73–1.61)
Other	‐	‐	‐
Ethnicity			
White	0.99 (0.60–1.65)	0.88 (0.54–1.44)	1.01 (0.59–1.73)
Other	‐	‐	‐
Born in the UK			
Yes	1.01 (0.63–1.62)	1.55 (0.96–2.52)	1.63 (0.99–2.71)
No	‐	‐	‐
Area of residence			
Suburb/town/rural	1.05 (0.73–1.50)	1.23 (0.87–1.74)	1.28 (0.90–1.84)
City	‐	‐	‐
Education			
No post‐secondary education	1.23 (0.86–1.75)	1.39 (0.99–1.94)	0.98 (0.70–1.38)
Post‐secondary education	‐	‐	‐
Religion			
Atheist or agnostic	0.84 (0.60–1.19)	0.83 (0.60–1.15)	0.75 (0.54–1.04)
Any religion	‐	‐	‐
Living alone			
No	0.97 (0.59–1.60)	1.15 (0.72–1.85)	0.81 (0.51–1.29)
Yes	‐	‐	‐
Children in the household			
No	1.08 (0.74–1.58)	1.19 (0.83–1.71)	1.75 (1.20–2.56)**
Yes	‐	‐	‐
Chronic health condition			
No	1.01 (0.64–1.60)	1.01 (0.66–1.56)	1.05 (0.68–1.63)
Yes	‐	‐	‐
Depression (PHQ‐9) caseness			
No	0.81 (0.49–1.35)	0.80 (0.49–1.31)	1.24 (0.74–2.09)
Yes	‐	‐	‐
Anxiety (GAD‐7) caseness			
No	1.05 (0.64–1.72)	1.11 (0.68–1.80)	0.85 (0.51–1.40)
Yes	‐	‐	‐
COVID‐19 PTSD caseness			
No	0.79 (0.50–1.25)	0.85 (0.54–1.33)	0.87 (0.54–1.40)
Yes	‐	‐	‐
Mental health treatment			
Current/past mental health treatment	1.14 (0.77–1.65)	1.28 (0.89–1.84)	1.16 (0.80–1.69)
Other	‐	‐	‐
Loneliness caseness			
No	0.82 (0.56–1.19)	0.81 (0.57–1.16)	0.71 (0.49–1.02)
Yes	‐	‐	‐
Neuroticism	1.04 (0.93–1.16)	0.99 (0.90–1.11)	0.99 (0.89–1.11)
Resilience	0.98 (0.94–1.03)	0.98 (0.94–1.03)	0.99 (0.94–1.03)
Somatisation	0.98 (0.94–1.01)	0.94 (0.91–0.97)***	0.93 (0.90–0.96)***
Paranoia	0.95 (0.91–0.99)*	0.95 (0.92–0.99)*	0.95 (0.92–0.99)*
Intolerance of uncertainty	1.01 (0.98–1.03)	1.02 (0.99–1.04)	1.04 (1.01–1.06)**
Death anxiety	1.01 (0.99–1.03)	1.00 (0.99–1.02)	0.99 (0.99–1.01)
COVID‐19 anxiety	0.99 (0.99–1.00)	1.00 (0.99–1.01)	0.99 (0.99–1.00)

^a^ Six participants classified as ‘Other gender’ not included due to low cell count. *p < 0.05; **p < 0.01; ***p < 0.001. C19PRC‐UKW1: baseline survey (March 2020).

Of the 853 additional respondents who were also eligible to be recontacted at C19PRC‐UKW4 having only entered the panel at the previous wave (C19PRC‐UKW3), 525 (61.5% recontact rate) were successfully followed up.

Table 3 compares the sociodemographic characteristics of responders and non‐responders at C19PRC‐UKW4: higher proportions of females, younger adults (aged 18–34 years), adults born outside the UK, adults not living alone, and adults living in cities were lost to follow‐up, compared to those who were re‐surveyed.

**TABLE 3:** Attrition analysis for Wave 4 of the COVID‐19 Psychological Research Consortium (C19PRC) Study (November–December 2020)

Respondent characteristic	C19PRC‐UKW4		*χ* ^2^ (*df*), *p*
	Responders (*N* = 1796) *N* (%)	Non‐responders (*N* = 1082) *N* (%)	
Gender			
Male	875 (48.7%)	468 (43.3%)	16.364 (2), <0.001
Female	919 (51.2%)	605 (55.9%)	
Other	2 (0.1%)	9 (0.8%)	
Age group (years)			
18–24	131 (7.3%)	290 (26.8%)	308.175 (5), <0.001
25–34	316 (17.6%)	277 (25.6%)	
35–44	344 (19.2%)	198 (18.3%)	
45–54	395 (22.0%)	153 (14.1%)	
55–64	337 (18.8%)	97 (9.0%)	
65+	273 (15.2%)	67 (6.2%)	
2019 household income			
≤£15.490	363 (20.2%)	223 (20.6%)	4.665 (4), >0.05
£15,491–£25,340	330 (18.4%)	216 (20.0%)	
£25,341–£38,740	347 (19.3%)	226 (20.9%)	
£38,741–£57,903	396 (22.0%)	207 (19.1%)	
≥£57,931	360 (20.0%)	210 (19.4%)	
Economic activity			
Employed	1150 (64.0%)	718 (66.4%)	1.606 (1), >0.05
Other	646 (36.0%)	364 (33.6%)	
Birthplace			
Born in UK	1642 (91.4%)	932 (86.1%)	19.991 (1) <0.001
Born elsewhere	154 (8.6%)	150 (13.9%)	
Household characteristics			
Single adult household	377 (21.0%)	173 (16.0%)	10.929 (1), <0.01
Other	1419 (79.0%)	909 (84.0%)	
Children under 18 years in household	497 (27.7%)	371 (34.3%)	14.030 (1) <0.001
Other	1299 (72.3%)	711 (65.7%)	
Place of residence			
Suburb/town/rural	1398 (77.8%)	738 (68.2%)	32.742 (1), <0.001
City	398 (22.2%)	344 (31.8%)	
Physical health			
Chronic health condition	648 (36.1%)	384 (35.5%)	0.102 (1), >0.05
None	1148 (63.9%)	698 (64.5%)	

### 3.2 Weight procedure Phase 1 longitudinal panel from baseline

The raking procedure successfully re‐balanced the characteristics of responders at this fourth wave (*N* = 1271) to the baseline proportions for gender (rebalance within 1%), age (exact rebalance), household income (within 0.4%), household composition (exact rebalance), and urbanicity (exact rebalance), ethnicity (within 0.8%), and born or raised in the UK (within 0.8%) – see Table S1. Applying this weight for all analyses of the C19PRC‐UKW4 survey data completed by this longitudinal panel (from baseline) is recommended to account for attrition over survey waves on core study outcomes.

### 3.3 Recruitment of new respondents: Phase 2

The median survey completion time for Phase 2 was 34 min 48 s. At Phase 2, 3073 adults were successfully engaged by Qualtrics partners and, following quality control checks, 1002 respondents were removed due to a failure to (1) complete the survey in full (*n* = 344); (2) satisfy the inclusion criteria (*n* = 185); (3) fulfil the legitimate respondent status (*n* = 50), or (4) satisfy country of residence sampling quotas (*n* = 420), or due to other minor technical errors (*n* = 3). This resulted in a Phase 2 sample of2071^1^, of which 292 respondents were recruited to ‘top‐up’ quotas due to attrition in Phase 1, and the remaining 1779 constituted the UK‐nation oversample. The ‘top‐up’ quotas successfully re‐balanced the C19PRC‐UKW4 cross‐sectional sample to be presentative of the UK adult population aged 18 years and older, with respect of age, gender, and household income (see Table S2).

### 3.4 Socio‐demographic and mental health characteristics of at C19PRC‐UKW4 respondents: Phases 1 and 2

Table 4 displays the socio‐demographic characteristics and prevalence of mental health conditions for the entire C19PRC‐UKW4 sample (*N* = 3867), stratified by Phase 1 and Phase 2, surveyed 9 months into the COVID‐19 pandemic in the UK. Participants varied across the strands in relation to gender, age, household income, economic activity, ethnicity, birthplace, children in household, and the prevalence of common mental disorders. The necessity to recruit to baseline quotas ensured the ‘top‐up’ strand had the highest proportion of females and younger adults, and the prevalence of all common mental disorders were highest in this strand (depression; 43.2%; anxiety 32.9%; and PTSD; 29.8%). Members of the longitudinal panel recruited at baseline had lower prevalence estimates for depression (25.3%) and anxiety (19.1%) at C19PRC‐UKW4; members of the longitudinal panel returning for the first time at this fourth wave and respondents in the booster oversample had higher, but similar, proportions of depression (30.5% vs. 29.7%) and anxiety (23.2% vs. 22.3%). Prevalence estimates for PTSD differed across the survey strands and was lowest for the oversample (16.0%), followed by the baseline longitudinal panel (16.9%) and the longitudinal panel first entering at the previous wave (21.1%).

**TABLE 4:** Socio‐demographic characteristics and prevalence of mental health disorders of the C19PRC‐UKW4 combined sample (*N* = 3867), stratified by phase of recruitment (November‐December 2020)

Respondent characteristic	Phase 1 (*N* = 1796) – longitudinal panel		Phase 2 (*N* = 2071) – new entrants		*χ* ^2^ (*df*) *p*
	From C19PRC‐UKW1 (*N* = 1271) *N* (%)^a^	From C19PRC‐UKW3 (*N* = 525) *N* (%)	Quota top‐ups (*N* = 292) *N* (%)	UK‐nation booster sample (*N* = 1779) *N* (%)	
Gender					
Male	603 (47.4%)	240 (45.7%)	127 (43.5%)	856 (48.1%)	18.0538 (12) *p* < 0.01
Female	668 (52.6%)	285 (54.3%)	160 (54.8%)	912 (51.3%)	
Other categories	0	0	5 (1.7%)	11 (0.6%)	
Age group (years)					
18–24	126 (9.9%)	50 (9.5%)	88 (30.1%)	128 (7.2%)	386.4239 (15) *p* < 0.0001
25–34	244 (19.2%)	128 (24.4%)	98 (33.6%)	222 (12.5%)	
35–44	225 (17.7%)	130 (24.8%)	30 (10.3%)	283 (15.9%)	
45–54	260 (20.4%)	101 (19.2%)	30 (10.3%)	296 (16.6%)	
55–64	226 (17.8%)	72 (13.7%)	28 (9.6%)	436 (24.5%)	
65+	191 (15.0%)	44 (8.4%)	18 (6.2%)	414 (23.3%)	
2019 household income					
£0–£15,490	286 (22.5%)	100 (19.0%)	56 (19.2%)	399 (22.4%)	96.9931 (12) *p* < 0.0001
£15,491–£25,340	239 (18.8%)	103 (19.6%)	80 (27.4%)	420 (23.6%)	
£25,341–£38,740	239 (18.8%)	113 (21.5%)	79 (27.1%)	450 (25.3%)	
£38,741–£57,930	261 (20.6%)	118 (22.5)	39 (13.4%)	340 (19.1%)	
£57,931+	246 (19.4%)	91 (17.3%)	38 (13.0%)	170 (9.6%)	
Economic activity					
Employed (full or part‐time)	831 (65.3%)	350 (66.7%)	192 (65.8%)	950 (53.4%)	61.397 (3) *p* < 0.0001
Other	440 (34.7%)	175 (33.3%)	100 (34.2%)	829 (46.6%)	
Ethnicity					
White	1170 (92.1%)	456 (86.9%)	255 (87.3%)	1729 (97.2%)	98.4482 *p* (3) <0.0001
Other	101 (7.9%)	69 (13.1%)	37 (12.7%)	50 (2.8%)	
Birthplace					
Born in UK	1177 (92.6%)	465 (88.6%)	258 (88.4%)	1683 (94.6%)	30.7279 *p* < 0.0001
Born elsewhere	94 (7.4%)	60 (11.4%)	34 (11.6%)	96 (5.4%)	
Household characteristics					
Single adult household	289 (22.7%)	109 (20.8%)	76 (26.0%)	422 (23.7%)	3.4863 *p* = 0.322544
Other	982 (77.3%)	416 (79.2%)	216 (74.0%)	1357 (76.3%)	
Children under 18 years living in household	311 (24.4%)	156 (29.7%)	74 (25.3%)	376 (21.1%)	17.8156 *p* < 0.001
Other	960 (75.6%)	369 (70.3%)	218 (74.7%)	1403 (78.9%)	
Mental health conditions					
Depression ‐ PHQ‐9 caseness	321 (25.3%)	160 (30.5%)	126 (43.2%)	528 (29.7%)	37.509 *p* < 0.0001
Not met	950 (74.7%)	365 (69.5%)	166 (56.8%)	1251 (70.3%)	
Anxiety ‐ GAD‐7 caseness	243 (19.1%)	122 (23.2%)	96 (32.9%)	396 (22.3%)	26.616 *p* < 0.0001
Not met	1028 (80.9%)	403 (76.8%)	196 (67.1%)	1383 (77.7%)	
PTSD caseness met	215 (16.9%)	111 (21.1%)	87 (29.8%)	285 (16.0%)	36.6838 *p* < 0.0001
Not met	1056 (83.1%)	414 (78.9%)	205 (70.2%)	1494 (84.0%)	

^a^ Weighted %.

In a final set of supplementary analyses (see Table S3), summary statistics for core political variables in the C19PRC‐UIKW4 study stratified by country are presented to further highlight the potential for robust between‐country socio‐political analyses in the context of the COVID‐19 pandemic using the C19PRC‐UKW4 study data.

## 4 DISCUSSION

By December 2020, four survey waves had been conducted for the C19PRC Study in the UK since its inception at the start of the COVID‐19 pandemic in March 2020. The C19PRC Study comprises a diverse sample and contains a huge array of mental health, psychological, socio‐economic, and political measures. The major objective of the C19PRC Study is to explain changes in UK adults' attitudes, experiences, and behaviours throughout the course of the COVID‐19 pandemic using a range of innovative measures and approaches.

Our Consortium has previously contributed to the on‐going debate about the strengths and limitations of probability versus non‐probability survey designs during the pandemic (McBride et al., 2020, 2021). Here, we focus our efforts on unpacking the core credentials of this ongoing longitudinal panel study by winter 2020: (1) the successful re‐interviewing approximately six‐in‐ten (62.8%) of baseline respondents at this fourth wave; (2) the low levels of attrition – only 15% of baseline respondents were completely lost to follow‐up by this stage in the study; and (3) the ability to successfully re‐engage ‘temporary dropouts’ (i.e. those adults who participate in some, but not all, waves) back into the panel at this fourth wave.

These outcomes from the C19PRC Study compare favourably to similar studies. For example, between April and September 2020, the US COVID‐19 Outbreak Public Evaluation (COPE) Initiative, a large, online longitudinal study (*N* = 6548, baseline sample) which assesses public attitudes, behaviours, and beliefs relating to the pandemic, and evaluates mental and behavioural health during the pandemic, interviewed participants four times. Participation declined from 51.9% at the first follow‐up to 28.5% at the third follow‐up survey (Czeisler et al., 2021b). Moreover, complete dropout from the survey was high; 57.6% of baseline respondents completed only that single survey wave (Czeisler et al., 2021b). We concur with concerns raised by (Czeisler et al., 2021a) that problems relating to retention of respondents over time tempers optimism and confidence in findings emerging from some longitudinal mental health surveys conducted during the pandemic. Evidence indicates that attempts to re‐engage ‘temporary dropouts’ is important to increase sample variability with respects to life changes (Müller & Castiglioni, 2020). Our Consortium has worked proactively at each survey wave post‐baseline to minimise study limitations due to sample attrition. Given the unpredictable nature of the COVID‐19 pandemic, and the likely impact that the pandemic has had on respondents' ability to participate at different waves, we continue to attempt to re‐engage all baseline respondents at each planned survey wave (of which there are two due to take place before the current ESRC funding grant ends in November 2021) so that they continue to have an opportunity to re‐enter the panel at a suitable time for them.

Analyses presented here revealed that attrition in the C19PRC Study longitudinal panel has mostly been influenced by baseline socio‐demographic characteristics as opposed to baseline experiences of mental health problems; that is, more women, younger adults, lower income earners, and those with dependent children have been lost‐to‐follow‐up over the four waves of data collection. Again, using the COPE Initiative as a comparison, attrition analyses in that study revealed that respondents who completed two or more of the four surveys administered between April and September 2020 had significantly lower prevalence estimates of adverse mental health problems at baseline compared to those not followed‐up. In the C19PRC Study, we found that although baseline mental health status for common conditions such as depression and anxiety did not predict attrition over subsequent waves (only lower levels of paranoia and somatization, and higher levels of intolerance of uncertainty, had a very small effect size in predicting participation), we demonstrated that, 9 months into the COVID‐19 pandemic in the UK, current (i.e. during the past 2 weeks) prevalence estimates of depression and anxiety (and to a lesser extent, PTSD) were lower for participants in the longitudinal panel returning from baseline, compared to other strands of the panel participating in this wave. Thus, this evidence suggests that, compared to those how have dropped out, adults who continue to participate in C19PRC surveys post‐baseline are likely to be doing better overall during the pandemic.

To address the issue of attrition head‐on, our Consortium adopted a strategy to conduct quota sampling replenishment of adults at post‐baseline waves according to specific socio‐demographic characteristics (i.e. gender, age, and household income) ascertained at baseline in an attempt to ‘re‐balance’ the sample to be nationally representative of the UK adult population at each survey wave. In practice, this strategy assumes that the pandemic experiences of adults entering the C19PRC Study post‐baseline to fill ‘vacant’ quotas are similar to those lost to follow‐up, although we are unable to test this assumption directly. Given the inequalities produced by the pandemic over a considerable period of time, it is possible that these sample replenishment procedures are not fool proof. However, we have demonstrated here that prevalence estimates for common mental health conditions for new entrants recruited at this fourth wave to replenish baseline quotas due to attrition (i.e. targeted recruitment of younger adults, more women, and lower income earners) were higher than those for members of the longitudinal panel (i.e. returning respondents). This evidence provides some reassurance that if attrition is not completely at random (i.e. the probability of missingness on the core mental health outcome variables in the C19PRC Study at this fourth wave is linked to respondent characteristics such as socio‐demographic factors) that the replenishment of the sample according to baseline quotas is successfully recruiting adults in these population groups who are experiencing poorer levels of mental health.

We also take this opportunity to highlight some additional limitations of the C19PRC Study design. Our inclusion criteria of restricting recruitment into the study to English‐speaking adults, whilst pragmatic to facilitate the prompt set‐up of the study during the rapidly unfolding pandemic in March 2020, precludes any analyses relating to how the pandemic has impacted members of the population not fitting into these categories. This being noted, we have collected detailed information on ethnicity and nationality over the course of the C19PRC Study, and we have demonstrated that our baseline sample was representative of the UK population with respect to both characteristics (McBride et al., 2020). The C19PRC Consortium also has an auxiliary two‐wave study of young people aged 13–24 years (*N* = 2002) which assess the impact of the pandemic on the health and relationships of this segment of the UK population (Levita et al., 2021a, 2021b). Analyses of this important study data is on‐going.

In summary, and in recognition of these recruitment outcomes and attrition analyses, we seek to offer guidance as to how the dynamic C19PRC Study data can be used to address specific COVID‐19 related research questions: (1) the amalgamation of data from specific strands of the survey (e.g. returning panels and ‘top‐up’ strand) produces a large, cross‐sectional survey (*N* = 2088), which is nationally representative of UK adults aged 18 years and older with respect to gender, age, and household income, and can be used to address point‐in‐time COVID‐19 related research questions; (2) analysis of data produced by the longitudinal panel returning from baseline (*N* = 1271) applying the C19PRC‐UKW4 weighting variable provides an optimal vehicle for the pursuit of research questions relating to changes in multiple aspects of health, wellbeing, and life experiences over the first 9 months of the pandemic; and (3) combining data across the all strands from C19PRC‐UKW4 will provide a large cross‐sectional sample (*N* = 3867) with good representation from all four UK nations to facilitate robust between‐country comparisons to assess how historical political events are impacting on the ability of adults living in across the UK to respond to, and cope with, the on‐going psychological demands and the existential threat of COVID‐19. The availability of geospatial data also facilitates the enrichment of individual survey responses via linkage to country‐specific external data resources (e.g. measures of area‐level deprivation; population density; availability of green spaces; area‐level rates of COVID‐19 testing/infection/death, etc.).

Consistent with UKRI ESRC funding regulations, the C19PRC‐UK study will be lodged with the UK Data Service before the end of 2021. In the interim, the baseline (C19PRC‐UKW1; March 2020) and first and second follow‐up (C19PRC‐UKW2, April–May 2020; C19PRC‐UKW3, July–August 2020) surveys are publicly available on the Open Science Framework (see https://osf.io/9emvp/). This will facilitate the public sharing of this rich data resource with scholars, academics, researchers, and stakeholders across a wide range of fields and disciplines.

## CONFLICT OF INTEREST

All authors declare no conflict of interest.

## Supporting information

Supporting Information S1

Supporting Information S2

## Data Availability

The baseline (C19PRC‐UKW1; March 2020) and first three follow‐up surveys (C19PRC‐UKW2, April–May 2020; C19PRC‐UKW3, July‐August 2020; C19PRC‐UKW4, November‐December 2020) surveys are publicly available on the Open Science Framework (OSF) (see https://osf.io/9emvp/). Data from subsequent waves will be uploaded to OSF and the UK Data Service in early 2022. In the interim, the data that support the findings of this study are available from the corresponding author upon reasonable request.

## ENDNOTES

^1^
49 participants were incorrectly recruited into Phase 2 ‘oversample’ strand, when they should have been a Phase 1 ‘re‐contact’ respondent. These respondents have since been re‐categorised correctly, but missing data exist for these participants on specific measures administered to Phase 1 respondents.
