# Supplementary material for: Design, content, and fieldwork procedures of the COVID‐19 Psychological Research Consortium (C19PRC) Study – Wave 4
Source: Int J Methods Psychiatr Res. 2021 Nov 5;31(1):e1899. doi: 10.1002/mpr.1899 (PMC8646695; doi:10.1002/mpr.1899)
Supplement: Supplementary file 1 — Supporting Information S1 [file MPR-31-e1899-s001.docx]

Design, content, and fieldwork procedures of the COVID-19 Psychological Research Consortium (C19PRC) Study – Wave 4

**Supplementary Measures Material**

Orla McBride^1*^, Sarah Butter^2^, Jamie Murphy^1^, Mark Shevlin^1^, Todd K. Hartman^2^, Kate M. Bennett^3^, Thomas VA Stocks^2^, Alex Lloyd^4^, Ryan McKay^4^, Jilly Gibson-Miller^2^, Liat Levita^2^, Liam Mason^5^, Anton P. Martinez^2^, Philip Hyland^6^, Frédérique Vallières^7^, Thanos Karatzias^8^, Carmen Valiente^9^, Carmelo Vazquez^9^, & Richard P. Bentall^2^

^1^ Ulster University, Northern Ireland

^2^ University of Sheffield, England

^3^ University of Liverpool, England

^4^ Royal Holloway, University of London, England

^5^ University College London, England

^6^ Maynooth University, Republic of Ireland

^7^Trinity College Dublin, Republic of Ireland

^8^Napier University, Scotland

^9^Complutense University of Madrid

Short title: C19PRC Study Wave 4 protocol

**Corresponding author:** Dr Orla McBride, School of Psychology, Ulster University, Cromore Road, Coleraine, BT52 1SA, Northern Ireland. Email: [o.mcbride@ulster.ac.uk](mailto:o.mcbride@ulster.ac.uk); Tel: 0044(0)2870123987.

**2. Methods**

**2.2 Measures**

Where appropriate/relevant, details are presented about the use of standardised and validated measures in this wave. Details are also provided where established/existing measures have been modified slightly for the purposes of collecting data during the pandemic. Unless otherwise stated, all other measures were derived by the C19PRC Study team for the purpose of collecting bespoke pandemic-specific data from survey respondents.

**2.2.1 Socio-demographic characteristics.** A detailed account of respondents’ socio-demographic history was collected (note, for this section, indicators marked ¥ are asked only of Phase 2 respondents given that this information was collected at earlier waves for Phase 1 respondents): *age* (18-24 years; 25-34 years; 35-44 years; 45-54 years 55-64 years; 65 years and over), *gender* (male, female, other), *ethnicity* (White British/Irish*; White non-British/Irish; Indian; Pakistani; Chinese; Afro-Caribbean; African; Arab; Bangladeshi; Other Asian; Other -specify – * further categorisation was sought from respondents as follows: White British; White English; White Welsh; White Scottish; White Northern Irish; or White Irish); *legal marital or same-sex status* (never married and never registered same-sex civil partnership (Single); separated, but still legally married; divorced; widowed; married; in a registered same-sex civil partnership; separated but still legally in a same-sex civil partnership; or formally in a same-sex civil partnership which is now legally dissolved; surviving partner from a same-sex civil partnership); *country of residence* (England, Scotland, Wales, Northern Ireland, Crown Dependencies – Channel Islands/Isle of Man), *country of birth* (England, Scotland, Wales, Northern Ireland, Crown Dependencies – Channel Islands/Isle of Man or somewhere else – specify country); *urbanicity* (living in a city, a suburb, a town or a rural area); *currently living in the same area where you grew up* (yes/no); *highest level of education*¥ (no qualifications; O-level/GCSE or similar; A-level or similar; diploma; undergraduate degree; postgraduate degree; technical qualification; or Other-specify); and *religion*¥ (Christian; Muslim; Jewish; Buddhist; Sikh; Atheist; Agnostic; or other-specify).

*Working class status*. Respondents were asked to self-report their working class status with a single-item: “*Would you consider yourself*?” (1) working class; (2) middle class; (3) upper class or (4) I don’t think of myself as belonging to any particular class.

*Social rank* (MacArthur Scale of Subjective Social Status; Adler, Epel, Castellazzo, and Ickovics (2000)). The MacArthur Scale of Subjective Social Status was developed to capture individuals’ sense of their place in the social ladder which accounts for multiple dimensions of socio-economic status (education, income, and occupation) and social position. Using a pictorial format, respondents were presented with a social ladder and asked to consider the following statement: “*Think of a ladder representing where people stand in the United Kingdom. At the top of the ladder are the people who are the best off – those who have the most money, the most education, and the most respected jobs. At the bottom are the people who are the worst off – those who have the least money, least education, and the least respected jobs or no job. The higher up you are on the ladder, the closer you are to the people at the very top; the lower you are, the closer you are to the people at the very bottom*”. *Please click the number below to show where you think you stand at this time in your life, relative to other people in the UK. 10-rungs.*

**2.2.2 Economic activity**

*2.2.2.1 Employment status.* Respondents were asked to indicate whether they were: employed full-time, employed part-time; self-employed (full-time), self-employed (part-time), been placed on the government ‘furlough’ scheme; unemployed, but looking for work; unemployed, looking after family or home; unemployed, long-term sick or disability full-time student; or retired. Respondents who indicated they were unemployed, but looking for work were asked “*Are you unemployed as a consequence of the coronavirus pandemic?*” (Yes/No).

*2.2.2.2 Keyworker status.* All respondents who indicated that they were not unemployed, a full-time student, or retired, were also asked to consider a definition of a ‘key worker’ (i.e. people whose jobs are vital to public health and safety during the coronavirus lockdown) and to determine whether their occupation was covered by any of the following categories (UK Cabinet Office/Department of Education, 2020): (1) Health and social care worker (e.g. all NHS staff including administrative and cleaning staff, care home workers); (2) Education and child care (e.g. nursery care workers and teachers); (3) Food and other necessary goods (e.g. staff involved in production, processing, distribution, sale and delivery of goods); (4) Key public services (e.g. postal workers, those required to run the justice system, religious staff, those responsible for managing the deceased and journalists providing public service broadcasting); (5) Local and national government (e.g. staff in administrative roles essential to the effective delivery of the COVID-19 response or delivering essential public services including payment of benefits); (6) Utility workers (e.g. staff needed to keep oil, gas, electricity, water and sewerage operations running, staff in the civil nuclear, chemical and telecom communications sectors); (7) Public safety and national security (e.g. police and support staff, Ministry of Defence civilian staff and armed forces personnel, fire and rescue staff, and workers responsible for border security, prisons, and probation); or (8) Transport (e.g. staff keeping air, water, road and rail passenger and freight transport modes operating).

*2.2.2.3 Underemployment* (Bell and Blanchflower (2021)). All respondents, regardless of current employment status, were administered the Bell-Blanchflower Unemployment Measure, which is a measure that assesses how well the labour force is being used in terms of skills, experience, and availability to work. Respondents were asked to consider “*Whatever your employment status, we would like now to ask you to respond to the following questions relating to hours of work*.” and answer the following questions (1) *how many hours a week do you typically work?; and (2) how many hours would you like to work?* An additional question was added *“how many hours a week did you typically work before the pandemic?* in order to capture changes in respondents’ typical working hours over the course of the pandemic. Responses to all three questions were recorded via an open text to insert a whole number.

**2.2.3 Household finances***.*

*2.2.3.1 Household income.* Self-estimated gross annual household income for 2019 was collected from all respondent using the following categories (which were used for quota sampling): (1) £0-£15,490; (2) £15,491-£25,340; (3) £25,341-£38,740; (4) £38,741-£57,930; and (5) £57,931 or more.

*2.2.3.2 Receipt of benefits*. A single-item was generated to collection information as to whether respondents were in receipt of benefits, as follows: “*Are you currently in receipt of any government benefits (not including child benefits and state pension?*” (Yes/No response).

*2.2.3.3 Income changes during the pandemic.* Respondents were asked to “*Please estimate the percentage change (either increase or decrease) in their monthly household income compared to the average monthly income before the COVID-19*” with response recorded on a visual slider ranging from 100% Less to 100% More, centred (starting point) at No change. Respondents were also asked *“Are you making savings because of the COVID-19 pandemic (i.e. do you have more money at the end of the month)?”* and “*Are you using savings to help your household manage during the COVID-19 pandemic?”*, both scored using (1) yes, (2) no, or (3) Don’t know. Respondents were also asked *“Has your overall debt increased or decreased this month due to COVID-19?”,* with responses scored on a 5-point Likert scale ranging from (1) increased a lot, to (5) decreased a lot; respondents were also able to indicate a response of ‘I do not have debt’. Respondents also self-reported their anxiety levels relating to household finances, as follows: “*On balance, how much are you worried about the way that your household finances have been affected by the coronavirus pandemic so far?*”. Responses were recorded on a 10-point Likert scale ranging from 1, not at all worried to 10, extremely worried. Finally, respondents were asked to consider “*Looking forwards, do you expect your financial security to* (1) get worse, (2) stay about the same, or (3) get better?”

*2.2.3.4 Paying bills.* One question was adapted from the Eurobarometer Survey (Blanchflower & Clark, 2020) to assess adults’ difficulties with paying bills: “*During the last month, would you say you found it difficult to pay your bills?*, scored on a 4-point Likert scale ranging from (1) Not at all difficult to (4) Very difficult. The original question was asked with reference to the last 12 months, and scored on a 3-point Likert scale ranging from 1, never to 3, most of the time, but the stated adaptions were necessary to obtain recent data on respondents’ financial circumstances during the pandemic (i.e., to reflect that although a significant proportion of the population have experienced considerable economic hardship over the course of the pandemic, the income of many other households has largely been unaffected, or even improved, as a result of the pandemic).

*2.2.3.5 Food insecurity.* Two questions were adapted from the US House Pulse Survey 2020 (US Census Bureau, 2020) to measure respondents’ experience of food insecurity, as follows: *“Before the first lockdown in March 2020, which of these statements best described the food eaten in your household?”,* with four response options to choose from *-*(a) Enough of the kinds of food I/we wanted to eat; (b) Enough, but not always the kinds of food I/we wanted to eat; (c) Sometimes not enough to eat or (d) Often not enough to eat; and (2) *In the past month, which of these statements best describe the food eaten in your household?*, with the same four response options provided - (a) Enough of the kinds of food I/we wanted to eat; (b) Enough, but not always the kinds of food I/we wanted to eat; (c) Sometimes not enough to eat or (d) Often not enough to eat.

**2.2.4 Housing characteristics.**

*2.2.4.1 Household composition.* Respondents provided information as to whether they lived alone or, if they lived with other people, how many adults lived with them. Parental status and living situation (with children of any age) was ascertained for all respondents, by providing the following statements (tick all that apply): (1) I do not have any children; (2) I have a child/children under 18 years or age, and he/she/they primarily live with me in my household; (3) I have a child/children under 18 years of age, but he/she/they primarily live elsewhere; (4) I have a child/children aged 18 years or over, and he/she/they primarily live with me in my household; (5) I have a child/children aged 18 years or over, but he/she/they primarily live elsewhere; and (6) someone else’s child/children under 18 years of age lives with me in my household.

*2.2.4.2 Physical properties of place of residence*. Phase 2 respondents were asked to indicate: (1) the type of property in which they live (a flat, house or bungalow); (2) how many bedrooms are in the property (ranging from 0 bedrooms, a single-room dwelling, studio or flat to 5 or more); and (3) how long they have lived at the property (under 1 year, 1-2 years, 3-5 years, 6-10 years, or more than 10 years). This was followed by a question about housing tenure (response options: own outright; own with a mortgage; shared ownership; renting; living rent free; squatting; or other). All Phase 1 respondents were asked: “*Since you last completed this survey, have you moved home?*” (Yes/No response). Adults indicating that they had moved, were asked “*Which of these options best describes the reason for your move?”:* (1) upgraded to a bigger home; (2) downsized home, for financial reasons; (3) moved to attend university; (4) evicted from home; (5) moved into or out of partner’s home; (6) moved into or out of family home; (7) moved for work reasons; (8) downsized home – for convenience; or (9) other. Individuals who indicated that they had moved to a new house were also asked about their new home in relation to (1) property type, (2) number of bedrooms and (3) housing tenure.

**2.2.5 Health conditions***.*

*2.2.5.1* *Health conditions posing increased risk for COVID-19.* The UK government directed that living with a major underlying health conditions is a risk factor for experiencing more severe ill-health and even death upon contracting COVID-19. Phase 2 participants were asked whether they and members of their immediate family were living with any major underlying health conditions (e.g., lung conditions, heart disease, kidney disease, liver disease, conditions affecting the brain and nerves, diabetes, problems with your spleen, a weakened immune system) (Yes/No response). All respondents were asked whether they or their partner were pregnant at the time of the survey and, if so, how many weeks? Respondents were also asked whether any members of their immediate family were pregnant at the time of the survey (yes/no response).

*2.2.5.2 General health* (Contoyannis, Jones, & Rice, 2004). A single-item taken from the British Household Panel Survey asked respondents to considered “*Compared to someone your own age, would you say your health has on the whole been?,* with responses recorded on a 5-point Likert scale ranging from 1, poor to 5, excellent.

**2.2.6 COVID-19.** The baseline wave (C19PRC-UKW1) was conducted in mid-March 2020 at the beginning of the pandemic when COVID-19 was a new virus, no existing measure was available to assess the general population’s knowledge, attitudes, and behaviours (KAB) of the virus. In order to assess COVID-19 related KAB, measures developed for use in studies of other global pandemics, for example the 2003 SARS outbreak, the 2009 H1N1 flu pandemic and the 2013-16 Ebola virus pandemic, were consulted and assessed for suitability and adaptation, where possible. Reliable and trusted web sources in the UK (e.g., Public Health England, the National Health Service; NHS) and internationally (e.g. the Centre for Disease Control, the WHO) were also consulted for current, evidence-based knowledge and information relating to the clinical presentation and transmission of COVID-19. Details of the newly devised questions/measures are described below. For consistency of measurement across the study, these newly developed items are incorporated into follow-up waves, as appropriate/necessary, with modest adjustments depending on the most current public health guidance in the UK.

*2.2.6.1 Sourcing information about COVID-19 and level of trust in sources*. ***Phase 2 only***. Respondents were asked about (1) “*How much information about COVID-19 that they had obtained from a variety of sources, including newspapers, television, radio, internet websites, social media, their doctor, other health professionals, government agencies, and family or friends?”;* and (2) “*How much trust they had in the information they got from each source?”* Responses were scored on a 4-point Likert scale ranging from 1 ‘None/Not at all’ to 4 ‘A lot’.

*2.2.6.2 Confidence in response to COVID-19.* Respondents were asked their views as to how well they think each of the following institutions have handled the COVID-19 pandemic: (1) the UK parliament; (2) the UK government; (3) the devolved government in Wales; (4) the devolved government in Scotland; (5) the devolved government in Northern Ireland; (6) your local government (council or local authority); (7) the police; (8) the legal system; (9) political parties; (10) scientists; (11) doctors and other health professionals and (12) pharmaceutical companies. Responses were scored on a 4-point Likert scale ranging from 1 ‘poor’ to 4 ‘very well’, with an ‘I’m not sure’ response also presented.

*2.2.6.3 Anxiety and threat relating to COVID-19.* Respondents’ degree of specific anxiety about the COVID-19 pandemic was assessed using a single visual slider scale, ranging from 0 ‘not at all anxious’ on the left-hand side to 100 ‘extremely anxious’ on the right-hand side (centred at 50, somewhat anxious). Separately, respondents recorded their view on the statement, “*How personally threatened do you feel by COVID-19*?” using a visual slider scale ranging from 0, not at all threatened on the left-hand side to 100, extremely threatened on the right-hand side, centred at 50.

*2.2.6.4 Perceived risk of contracting COVID-19.* Respondents estimated on a visual slider (ranging from 0, no risk on the left-hand side to 100, great risk on the right-hand side, centred at 50, moderate risk) their perceived percentage risk of contracting COVID-19 within the next month. Next, respondents were asked to consider “*If you were to become infected (or reinfected) with COVID-19 within the next month, how severe do you think your symptoms would be?* “ and to record their response on a visual slider (ranging from 0, not severe on the left-hand side to 100, very severe on the right-hand side, centred at 50, moderately severe).

*2.2.6.5 Experiences of self-isolation.* Respondents were presented with a definition of self-isolation (‘*self-isolation means that you have COVID-19 symptoms, or if someone you live with has symptoms, you must not leave your home for between 7-14 days’*) and were asked whether they had self-isolated during the COVID-19 pandemic (Yes/No response).

Those with children under 18 years of age living in the household were also asked two additional questions in relation to experiences of self-isolation: (1) *Have one or more of the children in your household (under the age of 18) been asked to self-isolate because they have tested positive for COVID-19?*; and (2) *Have one or more of the children in your household (under the age of 18) been asked to self-isolate because one of their close contacts has tested positive for COVID-19?*”. Both statements scored using a yes/no/don’t know response scale.

*2.2.6.6 Experiences of COVID-19 infection - self.* Respondents were asked “*To the best of your knowledge, to date, have you been infected by COVID-19?*” (I’m not sure/yes/no/ response categories). Three additional statements were presented to those responding ‘I’m not sure’ to clarify their response, as follows: “I have had symptoms but I do not think I have been infected with the COVID-19 virus”, “I have not had symptoms but I still think I have been infected with the COVID-19 virus”, or “I have had symptoms and I think I may have been infected with the COVID-19 virus however I have not been tested” (selection of one option only permitted).

For those indicating a ‘yes’ response, a series of additional questions were asked. First, two statements were presented to clarify their testing history: “I have had symptoms and I think I have been infected with the COVID-19 virus, however I have not been tested” or “Yes, I have been tested for COVID-19 and the test was positive”. Phase 1 respondents were then asked to report (1) “How unwell did you feel?”, with responses recorded on a visual slider scale ranging from 0 ‘Not at all unwell’ to 100 ‘Extremely unwell’, centred at 50; and (2) whether they were admitted to hospital (Yes/No response).

*2.2.6.7 Testing for COVID-19 – other.* Respondents were asked (Yes/No/Not applicable response) whether anyone else from their household had been diagnosed with COVID-19 (confirmed by a test) and whether anyone from their extended family had been diagnosed with COVID-19 (confirmed by a test).

*2.2.6.8 COVID-19-related deaths.* Respondents were asked whether anyone close to them had died because of COVID-19, with response options including ‘Yes’, ‘No’ and ‘Unsure – not certain death was caused by COVID-19’.

*2.2.6.9 COVID-19 Risk behaviours.* Respondents were asked about their engagement in a list of seven daily activities in the past week and asked to indicate, on how many days (response scale 1 ‘Not at all’ to 5 ‘Every day’), they had: (1) left the house for food, health reasons or work; (2) exercised outside the house; (3) met up with friends or extended family outside home; (4) met up with friends or extended family inside someone’s home; (5) gathered in a group of more than 2 people in a park or public space; and (7) left the house to provide assistance to a vulnerable or elderly person.

*2.2.6.10 Knowledge of, and compliance with, COVID-19 Regulations*. Respondents were asked their views on government decisions around COVID-19 regulations. First, respondents were asked to consider the following statement “*Since the pandemic began, the regulations and restrictions relating to COVID-19 have varied from place to place. In your view, how easy has it been for you to understand the COVID-19 regulations and restrictions in your area as they relate to*”, and were provided with 11 scenarios as follows: (1) socialising, ‘bubbling’ and overnight stays; (2) indoor and outdoor gatherings; (3) medically vulnerable and older people; (4) travel and public transport; (5) face coverings; (6) GP and hospital attendance; (7) childcare; (8) school or university attendance; (9) hospitality (i.e. bars, restaurants, coffee shops, hotels); (10) leisure activities; and (11) work practices. Responses were scored on a 5-point Likert scale ranging from 1, very easy to 5, very difficult. Respondents were also asked “*How easy would you say it is to find information on the regulations in your area?*, with response recorded using the same scale. Next, respondents were asked to consider the extent to which (scored on a 4-point Likert scale ranging from 1, not well at all to 4, very well) each of the following groups of people are following the COVID-19 lockdown rules within the UK (e.g., staying at home, maintaining social distancing): (1) young people; (2) old people; (3) students; (4) families with children; (5) poor people; (6) wealthy people; and (7) migrants. With specific reference to their country of residence, respondents were asked: “*In general, what percentage of people in* [England/Wales/Scotland/Northern Ireland/Crown Dependencies] *do you think are adhering to government rules regarding lockdown?”* with answers recorded on a visual slider scale ranging from 0-100, centred at 50. Finally, respondents were asked to consider how important should each of the following factors be when government makes decisions about lockdown: (1) protecting people vulnerable to COVID-19; (2) keeping shops and businesses open; and (3) keeping schools and universities open, with all responses scored on the 7-point Likert scale ranging from 1, very unimportant to 7, very important.

*2.2.6.11 Views on current and future state of the COVID-19 crisis.*  Data was collected to assess respondents’ views, “*Do you believe that the worst of the COVID-19 crisis in the UK is…*” (1) behind us, (2) happening now, and (3) ahead of us. Respondents were also asked: “*Regarding the current wave of COVID-19 in the UK, to what extent you do oppose/support*: (1) closure of non-essential businesses; (2) stay-at-home orders; (3) mandatory wearing of face masks in public places; and (4) government aid to small business with responses recorded on a 5-point Likert scale ranging from (1) strongly oppose, to (5) strongly support.

*2.2.6.12 COVID-19 vaccine.* At the time this survey was designed, several COVID-19 vaccines were in the final stages of approval for use in clinical practice. During the fieldwork for C19PRC-UKW4, the Pfizer-BioNTech vaccine was approved for use on 2 December 2020 and the vaccination rollout commenced in the UK on 8 December 2020. The Oxford-AstraZeneca vaccine was anticipated to be approved/deployed in January 2021 (UK Government Coronavirus (COVID-19) in the UK, 2021). Two statements were presented to respondents to ascertain their views about these vaccines, as follows: (1) “M*ultiple vaccines for COVID-19 have now been developed. Will you take a vaccine for COVID-19 when it becomes available to you?” (*Yes, No, Maybe); and (2) “M*ultiple vaccines for COVID-19 have now been developed. Will you give your child a vaccine for COVID-19 when it becomes available?”* (Yes, No, Maybe, Not applicable – I don’t have children under 18).

All respondents were asked to report their views on COVID-19 vaccines in the UK. For this aim, respondents were presented with a statement “*Vaccines for COVID-19 that will be made available to the UK public will be...*” and asked to record their views on a visual slider scale, ranging from 0, completely disagree on the left-hand side to 100, completely agree on the right-hand side (centred at 50), in relation to (1) effective; (2) safe; (3) accessible; (4 affordable; and (5) accepted by the majority of the population. Finally, respondents were asked whether they agree (yes/no response) to this statement: “*The UK government should make it mandatory that citizens take a vaccine for COVID-19*.”

*2.2.6.12 COVID-19 related purchasing behaviours.* A series of questions were developed to measure respondents’ purchasing behaviours during the latest wave of the pandemic. Respondents were asked to report the extent to which they increased their purchasing of the following items in recent weeks because of the COVID-19 pandemic: (1) tinned food; (2) water; (3) sanitary products (e.g., hand sanitiser); (4) toilet roll; (5) dried foods (e.g., pasta. rice); (6) bread; (7) pharmacy products (e.g., painkillers, cold/flu products); (8) batteries; (9) fuel (heating or car fuel); and (10) alcohol. Responses were recorded on a five-point Likert scale ranging from (1) ‘not at all’ to 5 ‘very considerably’.

*2.2.6.13 Stock availability in supermarkets.* Respondents were asked to report their views (on a 5-point Likert scale ranging from 1, strongly disagree to 5, strongly agree) on anticipated stock availability in supermarkets in the future based on their experiences of supermarket shopping in the past week, as follows: (1) I expect supermarkets will take a long time to restock products; (2) I expect supermarkets are likely to run out of stock very soon; (3) I expect supermarkets will be well stocked when I arrive; (4) There is a short distances between my home to the supermarkets in my area; (5) During the first COVID-19 lockdown, it was easy for me to purchases items such as rice, tinned food, and toilet paper; (6) I have recently seen shortages in supermarkets nearby; (7) I have recently been told by somebody that there are shortages in supermarkets; and (8) I have recently seen supermarket shortages in the media.

*2.2.6.14 Food type consumption.* Respondents were asked to consider “*How important it is to them that the food you buy at the moment is*”: (1) healthy; (2) convenient to buy and prepare; and (3) affordable, with answers recorded on a 5-point Likert scale ranging from 1, not at all to 5, very important.

**2.2.7 Mental health.** Experiences of mental health difficulties are core outcomes for the C19PRC Study. A key objective of the study was to administer a range of brief, standardised questionnaires to screen for the presence of common mental disorder, which would be repeated across all survey waves. Details are included below.

*2.2.7.1* *Patient Health Questionnaire-9 (PHQ-9*) (Kroenke, Spitzer, & Williams, 2001). Depression was assessed with the PHQ-9, a nine-item measure which corresponds to the DSM-IV Diagnostic Criterion A symptoms for major depressive disorder (American Psychiatric Association, 2000). Participants were asked how often, over the last two weeks, they had been bothered by each of the depressive symptoms. Response options were “not at all”, “several days”, “more than half the days”, and “nearly every day”, scored as 0, 1, 2 and 3, respectively. PHQ-9 scores range from 0 to 27, with scores of ≥5, ≥10, ≥15, representing mild, moderate and severe levels of depression severity (Kroenke et al., 2001). A threshold of ≥10 was used in this study. Psychometric properties of the PHQ-9 are well documented (see Kroenke, Spitzer, Williams, and Löwe (2010) for an overview).

*2.2.7.2 Suicidality.* Following the PHQ-9, the last item of which asks respondents about whether they experienced thoughts of dead or self-harm in the last two weeks, respondents were asked if they would be content to answer a series of questions relating to thoughts and actions of self-harm and suicide (adapted from the 2014 English Adult Psychiatric Morbidity Survey (McManus, Bebbington, Jenkins, & Brugha, 2016)). Respondents who answered ‘No’ were automatically skipped to the next measures (GAD-7, see next sub-section); respondents who answered ‘Yes’ were presented with the following statement to assess lifetime suicidal ideation: “*There may be times in everyone's life when they become very miserable and depressed and may feel like taking drastic action because of these feelings. Have you ever thought of harming yourself or taking your life, even if you would not really do it*?” (Yes/No response). Next, the respondent was asked to re-consider the statement but with specific reference to the time period since the pandemic began (Yes/No response). Respondents were then asked, “*Have you ever made an attempt to take your own life*?” (Yes/No response). Filtering was imposed depending on the respondent’s answer: (1) adults who responded ‘No’ were asked if they had ever deliberately harmed themselves in any way but not with the intention of taking your own life (Yes/No response) and, if yes, was this in (a) the last two weeks and/or (b) in the last year but not in the last two weeks (Yes/No response to both questions); (2) adults who responded ‘Yes’ were asked if the attempt to take their own life happened in (a) the last two weeks and/or (b) in the last year but not in the last two weeks (Yes/No response to both questions), before being asked if they had ever deliberately harmed themselves At the end of the survey, respondents who reported that they had experienced lifetime suicidal thoughts, suicidal thoughts during the pandemic, having ever attempted suicide or having ever self-harmed, were presented with contact information for self-harm or suicide support services

*2.2.7.3 Generalized Anxiety Disorder Scale (GAD-7)* (Spitzer, Kroenke, Williams, & Löwe, 2006). Experiences of generalized anxiety were assessed using the GAD-7. Respondents were asked to report, on a 4-point Likert scale ranging from 1 (not at all) to 4 (nearly every day), how often in the past 7 days they were bothered by seven anxiety symptoms (e.g. trouble relaxing, becoming easily annoyed or irritable). The GAD-7 was originally validated in a primary care sample and a cut-off score of 10 had a sensitivity value of 0.89 and a specificity value of 0.82 for identifying generalised anxiety disorder (Spitzer et al., 2006), and a threshold of 10 was used in this study. The GAD-7 has demonstrated good reliability and construct validity, as evidenced by strong associations with other established measures of anxiety as well as diagnoses of GAD and its associations with depression, self-esteem, life satisfaction, and resilience (Löwe et al., 2008).

*2.2.7.4 International Trauma Questionnaire (ITQ)* (Cloitre et al., 2018). Post-traumatic stress disorder was assessed using the ITQ, a self-report measure of ICD-11 PTSD based on a total of six symptoms across the three symptom clusters of Re-experiencing, Avoidance, and Sense of Threat; each symptom cluster is comprised of 2 symptoms. Participants were asked to complete the ITQ as follows: “…in relation to your experience of the COVID-19 pandemic, please read each item carefully, then select one of the answers to indicate how much you have been bothered by that problem in the past month”. The PTSD symptoms are accompanied by three items measuring functional impairment caused by these symptoms. All items are answered on a 5-point Likert scale, ranging from 0 (Not at all) to 4 (Extremely) with possible PTSD scores ranging from 0 to 24. A score of ≥ 2 (Moderately) is considered ‘endorsement’ of that symptom. A PTSD diagnosis requires traumatic exposure, and at least one symptom to be endorsed from each PTSD symptom cluster (Re-experiencing, Avoidance, and Sense of Threat), and endorsement of at least one indicator of functional impairment. The psychometric properties of the ITQ scores have been demonstrated in multiple general population (Ben‐Ezra et al., 2018; Cloitre et al., 2019) and clinical and high-risk samples (Hyland et al., 2017; Karatzias et al., 2016; Vallières et al., 2018) samples.

*2.2.7.5 Persecution and Deservedness Scale* (PaDS) (Melo, Corcoran, Shryane, & Bentall, 2009). Paranoia was assessed with five items taken from the persecution subscale of the persecution and deservedness scale (PaDS), a measure designed for use with both clinical and population samples and which has been validated against both questionnaire and clinical measures of paranoia (Elahi, Algorta, Varese, McIntyre, & Bentall, 2017; Melo et al., 2009). Participants rated their agreement on a 5-point scale with statements such as “I’m often suspicious of other people’s intentions towards me” and “You should only trust yourself.” Response options ranged from 1 = strongly disagree to 5 = strongly agree. Scale reliability for the five items was very good (α = 0.84) in a previous epidemiological study of UK citizens (McIntyre, Wickham, Barr, & Bentall, 2018).

*2.2.7.6 Treatment seeking behaviour for mental health difficulties*. All respondents were asked about their history of mental health difficulties. The following statement was presented: “Mental health difficulties are very common. It will help us understand our survey results if you would tell us whether you currently or have in the past received treatment (medication or talking therapies) for these kind of difficulties”, along with the following response categories: (1) I never received treatment for mental health problems; (2) I have received treatment for mental health problems in the past; (3) I am currently receiving treatment for mental health problems; (4) I am currently receiving treatment for mental health problems, but it has been cancelled temporarily due to the lockdown; and (5) I’d prefer not to answer.

**2.2.8 Psychological factors.**

*2.2.8.1 Big-Five Inventory (BFI-10)* (Rammstedt & John, 2007). The five personality traits of openness to experience, conscientiousness, extroversion, agreeableness and neuroticism were assessed using the BFI-10, which contains items two items per personality construct such as ‘I see myself as someone who is reserved’, ‘ I see myself as someone who tends to be lazy’, and ‘I see myself as someone who has few artistic tendencies’. Rammstedt and John (2007) reported good reliability and validity for the 10-item scale.

*2.2.8.2 Loneliness Scale* (Hughes, Waite, Hawkley, & Cacioppo, 2004). Social connectedness was measured using the three-item Loneliness Scale, which was specifically designed for use in large-scaled population surveys (Hughes et al., 2004). Respondents were asked how often they felt: (1) that they lacked companionship; (2) left out; and (3) isolated from others. Responses were scored on a 3-point scale (hardly ever, sometimes, or often).

*2.2.8.3 Religious identity and beliefs.* An 9-item Monotheist and Atheist Beliefs Scale derived from a longer scale developed by the present authors (Alsuhibani, Shevlin, & Bentall, (in submission)) was included. This included items measuring religiosity, for example ‘*The soul is immortal*’ and atheism, for example, “*It is wrong to indoctrinate children into religion*”. Factor analysis of the longer scale indicated that religiosity and atheism are separate albeit negatively correlated constructs.

*2.2.8.4 Interpersonal Reactivity Index (IRI)* (Davis, 1980, 1983). **Phase 2 respondents only.** The IRI is by far the most widely used instrument to assess empathy (Pulos, Elison, & Lennon, 2004). The 28-item self-report measure consists of four, 7-item subscales (empathetic concern, personal distress, fantasy, and perspective-taking), which are measured on a 6-point Likert scale ranging from 1 ‘strongly disagree’ to 6 ‘strongly agree’. Two subscales of the IRI - perspective-taking (cognitive empathy) and empathic concern (affective empathy) - were administered to respondents. Examples of cognitive empathy included “*Before criticizing somebody, I try to imagine how I would feel if I were in their place*” and “*I believe that there are two sides to every question and try to look at them both*”, whereas examples of affective empathy items included “*I often have concerned feelings for people less fortunate than me*” and “*When I see someone being treated unfairly, I don't feel much pity for them*”. Both subscales have very good reliability (perspective-taking, α=0.79; emphatic concern, α=0.80) (Pulos et al., 2004).

*2.2.8.5 Conspiracy mentality scale (CMS)* (Imhoff & Bruder, 2014). Conspiracy mentality is a generalized political attitude, distinct from established generalized political attitudes like right-wing authoritarianism (RWA) and social dominance orientation (SDO) (Imhoff & Bruder, 2014). Respondents completed five items of the CMS (scored on a 5-point scale from 1 ‘Certainly not 0%’ to 11 ‘Certainly 100%’), including: ‘I think that many very important things happen in the world, which the public is never informed about’; ‘I think that politicians usually do not tell us the true motives for their decisions’; and ‘ I think that there are secret organizations that greatly influence political decisions’.

*2.2.8.6 Hopefulness (Brief-H-Pos Scale)* (Fraser et al., 2014). Respondents were asked to complete the Brief-H-Pos Scale, a two-item measure which is a positive re-framing of the negatively worded two-item Hopeless Scale (Everson et al., 1996). The two items, “The future seems to me to be hopeful and I believe that things are changing for the better” and “I feel that it is possible to reach the goals I would like to strive for”, are scored on a 5-point Likert scale ranging from 1, absolutely disagree to 5, absolutely agree (higher scores indicate higher levels of hopefulness). Fraser et al. (2014) demonstrated that the scale had good internal consistency, test-re-test reliability and concurrent validity (compared to the Beck Hopelessness Scale (Beck, Weissman, Lester, & Trexler, 1974) and the Centre for Epidemiological Studies on Depression Scale (Radloff, 1977)), and recommended the scale as a useful screener for hopefulness in large general population studies.

*2.2.8.9 Happiness* (Office for National Statistics, 2016). A single-item measuring subjective happiness, as included in the Annual Population Survey, was included, as follows: *“Overall, how happy did you feel yesterday, where 0 is ‘not at all happy’ and 10 is ‘completely’ happy?”* Scores of 0-4 are considered to reflect ‘low’ happiness, scores of 5-6 ‘medium’ happiness, 7-8 ‘high’ happiness’ and 9-10 ‘very high’ happiness, levels (Office for National Statistics, 2016).

*2.2.8.10 Life satisfaction.* A series of questions were generated to assess respondents’ satisfaction with life during the pandemic. First, respondents were asked “*Thinking about your life as it is right now, how satisfied are you with your life?*” and presented with a visual slider indicating ‘level of satisfaction’ ranging from 0 ‘Completely unsatisfied’ to 100 ‘Completely satisfied’ centred at 50. Next, respondents were asked to compare their lives before the pandemic to now and indicate whether they viewed their life as ‘better now’, ‘worse now’ or ‘no different’ than before the COVID-19 pandemic. Finally, respondents were presented with a list of 19 areas of life, including daily activities, such as home life, diet and exercise, relationships, work life, socialising, etc. and asked to indicate whether each area of life was ‘better, worse, or no different’ now than before the COVID-19 pandemic.

*2.2.8.11* *Short Warwick-Edinburgh Mental Well-being Scale (SWEMWBS)* (Stewart-Brown et al., 2009). The SWEMWBS is a 7-item unidimensional scale which has robust measurement properties for monitoring mental well-being in population surveys. Respondents answered the following questions with reference to the last two-weeks: (1) I’ve been feeling optimistic about the future; (2) I’ve been feeling useful; (3) I’ve been feeling relaxed; (4) I’ve been dealing with problems well; (5) I’ve been thinking clearly; (6) I’ve been feeling close to other people; and (7) I’ve been able to make up my own mind about things. All items are scored on 5-point Likert scale, ranging from 1, none of the time to 5, all of the time. Raw scores range from 7-35, with higher scores indicating overall better mental wellbeing. The total raw scores can then converted into a metric score using the following conversion table: <https://warwick.ac.uk/fac/sci/med/research/platform/wemwbs/using/howto/swemwbs_raw_score_to_metric_score_conversion_table.pdf>. Raw scores need to be converted into metric scores in order for comparisons to be made across different studies.

*2.2.8.12 Cognitive Reflection Task of Analytical Reasoning (CRT).* ***Phase 2 respondents only.*** *CRT* is a measure of analytical reasoning (Frederick, 2005). Respondents’ level of ability was assessed using an adapted version which included two additional items as well as the three in the original scale CRT. Respondents were asked to solve the following five problems, each of which is designed to stimulate intuitively appealing but incorrect responses: (1) A bat and a ball cost £1.10 in total. The bat costs £1.00 more than the ball. How much does the ball cost? (2) If it takes 5 machines 5 minutes to make 5 widgets, how long would it take 100 machines to make 100 widgets? (3) In a lake, there is a patch of lily pads. Every day, the patch doubles in size. If it takes 48 days for the patch to cover the entire lake, how long would it take for the patch to cover half of the lake? (4) If you’re running a race and you pass the person in second place, what place are you in? (5) A farmer had 15 sheep and all but 8 died. How many are left? Each problem hints at an incorrect answer and analytic reasoning (or ‘slow thinking’; (Kahneman, 2012)) reflects correct responses obtained by ignoring or discounting the hinted answer. The response format was multiple choice with three foil answers (including the hinted incorrect answer) as recommended by Sirota and Juanchich (2018).

*2.2.8.13* *Social engagement/contact*. **Phase 1 respondents only**. Two items were included to assess respondents’ social contact with family and friends during the pandemic(Seeman et al., 2011): (1) “How often are you in contact with any members of your family, that is, any of your brothers, sisters, parents, or children who do not live with you, including visits, phone calls, letters, or emails? and (2) How often are you in contact with any of your friends, including visits, phone calls, letters, or emails? Both statements were scored on an 8-point scale ranging from 1, never or hardly ever to 8, several times a day. With specific reference to the upcoming Christmas/holiday period, respondents were asked the extent to which they were worried (scored on a 10-point Likert scale ranging from 0, not at all worried to 10, extremely worried), about being able to physically visit with friends and relatives. Next, respondents were asked to gauge how difficult they think it will be to physically visit with friends and relatives during the upcoming Christmas holiday period (responses recorded on a 5-point Likert scale ranging from 1, not at all difficult to 5, very difficult).

**2.2.9 Socio-Political module.**

*2.2.9.1 Citizenship.* A single-item, adapted from the British Election Study (2019), was administered to collect data on respondents’ citizenship, as follows: “*Are you a citizen of any of the following (tick all that apply)*”: (1) the United Kingdom (Great Britain and Northern Ireland); (2) any member state of the European Union; (3) any commonwealth country; or (4) any other country.

*2.2.9.2 Language.* All respondents were asked “*Do you speak any of the following languages (tick all that apply)?”:* (1) Scots; (2) Ulster Scots; (3) Scots Gaelic; (4) Irish; (5) Welsh or (6) I don’t speak any of these languages. Those indicating that they spoke one of more of the languages presented, were asked with reference to their country of residence [England/Wales/Scotland/Northern Ireland/Crown Dependencies], “*Which of the following languages do you think should be recognised as an official language (tick all that apply)*?: (1) Scots; (2) Ulster Scots; (3) Scots Gaelic; (4) Irish; (5) Welsh, or (6) None.

*2.2.9.3 Voting behaviour.* **Phase 2 respondents** were asked to report their voting behaviour in response to the European Referendum (May 2016) (options voted Leave, voted Remain, did not vote, ineligible to vote). Respondents were also asked to indicate: the party to whom they gave their 1^st^ preference vote in the 2019 general election: (1) I was not eligible to vote; (2) I was eligible to vote but I did not vote, (3) Alliance Party of Northern Ireland; (4) BREXIT Party; (5) Conservative and Unionist Party; (6) Democratic Unionist Party (DUP); (7) Green Party; (8) Labour Party; (9) Plaid Cymru; (10) Scottish National Party (SNP); (11) Sinn Féin; (12) Social Democratic and Labour Party (SDLP); (13) UK Independence Party (UKIP); (14) Ulster Unionist Party; or (15) Other (specify). They were also asked the political party to which they most identify with the same political party response options as above, as well as a ‘None’ option.

Adapting a question from the British Election Study 2015 (Fieldhouse et al., 2016), respondent were asked to indicate, with reference to the political party with which they most identify: “When I speak about this party, I usually say ‘we’ instead of ‘they’” and “When people criticize this party, it feels like a personal insult” – responses to both questions were scores on a 5-point Likert scale ranging from (1) Strongly disagree to (5) Strongly agree. If respondents indicated that they did not identify with any political party, they skipped this section.

One additional question, adapted from the British Election Study 2017 Fieldhouse et al. (2018), asked respondents how they would describe their (1) political affiliation (on a 10-point scale ranging from 1 ‘left-wing’ to 10 ‘right-wing’).

*2.2.9.4 Social Dominance Scale* (SDO_7_) (Ho et al., 2015). Respondents’ levels of social dominance orientation were assessed using the eight-item SDO_7._ Respondents were asked the extent to which they opposed/favoured statements such as ‘*An ideal society requires some groups to be on top and others to be on the bottom’*, ‘*Some groups of people are simply inferior to other groups’*; and ‘*We should do what we can to equalize conditions for different groups’*. Response were scored using a 5-point Likert scale ranging from 1 ‘Strongly oppose’ to 5 ‘Strongly Favour’. Ho et al. (2015) demonstrated the SDO_7_ had good criterion and construct validity.

*2.2.9.5 Very Short Authoritarianism Scale (VSA)* (Bizumic & Duckitt, 2018). Under the Dual-Process Motivational Model (Duckitt, 2001, 2009), right-wing authoritarianism (RWA) and social dominance orientation (SDO) are conceptualised as value-attitude-belief dimensions which emerge from two different motivational schemas: threat-control (RWA) and competition-dominance (SDO). Both are robust predictors of a range of right-wing political beliefs, including prejudice. Past research also demonstrates that RWA can interact with the perception of threat to produce support for anti-democratic policies (Cohrs, Maes, Moschner, & Kielmann, 2007; Kossowska et al., 2011). The six-item VSA was used to assess respondents’ levels of RWA, and includes items such as: ‘*It’s great that many young people today are prepared to defy authority*’; ‘*What our country needs most is discipline, with everyone following our leaders in unity*’; and ‘*Our society does NOT need tougher government and stricter laws’*. All items were scored on a 5-point Likert scale ranging from 1 ‘strongly disagree’ to 5 ‘strongly agree’, with three items reverse coded.

*2.2.9.6 Attitude towards migrants*. Three items from the British Social Attitudes Survey (British Social Attitudes Survey, 2015) were used to assess respondents’ attitudes towards migrants, as follows: (1) “*Would you say it is generally bad or good for Britain's economy that migrants come to Britain from other countries*?” (scored on a 10-point scale ranging from 1 ‘extremely bad’ to 10 ‘extremely good’) (2) “*Would you say that Britain's cultural life is generally undermined or enriched by migrants coming to live here from other countries?”* (scored on a 10-point scale ranging from 1 ‘undermined’ to 10 ‘enriched’); and (3) “*Some migrants make use of Britain's schools, increasing the demand on them. However, many migrants also pay taxes which support schools and some also work in schools. Do you think that, on balance, migration to Britain reduces or increases pressure on the schools across the whole of Britain?”* (scored on a 5-point scale ranging from 1 ‘reduces pressure a lot’ to 5 ‘increases pressure a lot’)?

*2.2.9.7 Group conformity.* Two items were used in this wave to serve as a proxy measure for group conformity, which is expected to relate to right-wing authoritarianism. First, respondents were asked the extent to which they agree with the following statement about integration policies (single item adapted from the European Social Survey (2015): (1) “*Having a diverse mix of people in* [England/Wales/Scotland/Northern Ireland/Crown Dependencies] makes it a much better place”, (scored on a 5-point Likert scale ranging from 1, strongly disagree to 5, strongly agree). Second, using an item adapted from the 2019 American Trends Panel (ATP) (Menasce Horowitz, 2019), respondents were asked their view on whether “*It is better for* [England/Wales/Scotland/Northern Ireland/Crown Dependencies] *if almost everyone shares the same customs and traditions* (scored on a 5-point Likert scale ranging from 1, strongly disagree to 5, strongly agree).

2.2.9.8 *Populist Attitudes* (Castanho Silva, Jungkunz, Helbling, & Littvay, 2020). Populism, as described by Mudde (2004), is a thin-centred ideology according to which society is divided into two homogeneous and antagonistic groups (i.e., the ‘good people’ and the ‘corrupt elites’). Respondents were presented with this six-item scale, which measures three, two-item, constructs of ‘people-centrism’, ‘anti-elitism’ and ‘Manichaean outlook’, as follows: (1) “*Politicians should always listen closely to the problems of the people”*; (2) “*Politicians don’t have to spend time among ordinary people to do a good job*”*; (3) “*The government is pretty much run by a few big interests looking out for themselves”*; (4) “*Government officials use their power to try to improve people’s lives**”; (5) “*You can tell if a person is good or bad if you know their politics”*; and (6) “*The people I disagree with politically are just misinformed”*. All items are scored on a 5-point Likert scale ranging from 1, strongly disagree to 5, strongly agree (items marked * to be reverse coded). Castanho Silva et al. (2020) summarised that the six-item scale had high cross-national validity, and medium conceptual breadth and external validity.

*2.2.9.9 National belongingness.* Respondents were asked “*How much do you feel you belong to each of the following?*”: (1) Britain; (2) England; (3) Wales; (4) Scotland; (5) Northern Ireland; and (6) Ireland. Responses were recorded on a slider scale ranging from 0 ‘Do not belong at all’ to 10 ‘Very much belong’, centred at 5.

*2.2.9.10 National identity (*Adapted from Davidov (2011) – references to United States were replaced with the UK nations). Respondents were asked to record their level of identity (presented on a slider scale ranging from 0 ‘Do not identify at all’ to 10 ‘Very much identify’, centred at 5) for each of the following: (1) British; (2) English; (3) Welsh; (4) Scottish; (5) Northern Irish; and (6) Irish. Respondents were asked to report which of these identities was their most important identity but were also provided with a ‘None of the above’ option (and in this case, were not asked subsequent questions in relation to identity). Next, respondents were asked a series of questions relating to their most important identity only: (1) “*How proud are you of being* [British/English/Welsh/Scottish/Northern Irish/Irish]?; (2) “*The world would be a better place if people from other countries were more like the* [British/English/Welsh/Scottish/Northern Irish/Irish]” and (3) “*Generally speaking,* [Britain/England/Wales/Scotland/Northern Ireland/Ireland] *is a better country than most countries*”, with responses to all three statements scored on a 5-point Likert scale ranging from 1, strongly disagree to 5, strongly agree.

*2.2.9.11 Patriotism.*

*2.2.9.11.1 Symbolic patriotism.*  (Adapted from Huddy and Khatib (2007) – references to the United States were replaced with the UK nations). Symbolic patriotism represents a relatively abstract, affective attachment to a nation and its core values (Parker, 2010). Respondents were presented with an image of the national flag for each of the UK, England, Wales, Scotland, Ireland, and the European Union, and asked to indicate on a slider scale ranging from -5 Negative to 5 Positive (centred at 0, Neutral), “*How positive do you feel when you see each flag?*”.

*2.2.9.11.2 Constructive patriotism* (Adapted from Huddy and Khatib (2007) – three out of four items were included, and references to the United States were replaced with UK nations). Constructive patriotism can be regarded as the relationship an individual has with their country which is characterised by a more flexible identification, support for constructure criticism, and a desire to implement positive change (Schatz, 1994). Respondents were asked to answer the following items with reference to their *country of residence,* scored on a 5-point Likert scale ranging from strongly disagree to strongly agree: (1) *People should work hard to move* [England/Wales/Scotland/Northern Ireland/Crown Dependencies] *in a positive direction*; (2) *If I criticize* [England/Wales/Scotland/Northern Ireland/Crown Dependencies], *I do so out of love of country*; (3) *I oppose some policies of* [England/Wales/Scotland/Northern Ireland/Crown Dependencies] *because I care about it and want to improve it*.

*2.2.9.11.3 Uncritical Patriotism*. (Adapted from Huddy and Khatib (2007) - three out of seven items were included, and references to the United States were replaced with UK nations). Uncritical or ‘blind’ patriotism is characterised by an unwillingness both to criticize and accept criticism of one’s own nation (Huddy & Khatib, 2007). The following three items assessed uncritical patriotism (scored on a 5-point Likert scale ranging from strongly disagree to strongly agree): (1) I *support* [England/Wales/Scotland/Northern Ireland/Crown Dependencies]’s *leaders even if I disagree with their actions;* (2) *People who do not wholeheartedly support* [England/Wales/Scotland/Northern Ireland/Crown Dependencies] *should live elsewhere*; and (3) *I support* [England/Wales/Scotland/Northern Ireland/Crown Dependencies]’s *policies for the very reasons that they are the policies of my country.*

*2.2.9.12 Country thermometer (Adapted from* British Election Study (2019) – references to European countries such as Greece, Poland, etc., were replaced with UK nations). A single item was used to respondents’ view on the measure: “*How warm to do you feel toward the following*?: (1) English people; (2) Scottish people; (3) Welsh people; (4) Northern Irish people; (5) British people; and (6) Irish people. Responses were scored on a visual slider ranging from -5 (very cold) to 5 (very warm), centred at 0.

*2.2.9.13 Place Resentment* (Adapted from Munis (2020)). Nine items were used to assess respondents’ resentment, based on their country of residence: (1) [England/Wales/Scotland/Northern Ireland/Crown Dependencies] *gets less in resources compared to other parts of the UK*; (2) [England/Wales/Scotland/Northern Ireland/Crown Dependencies] *pays more in taxes to the UK than it gets back;* (3) *When we’re hit by bad times, people living in* [England/Wales/Scotland/Northern Ireland/Crown Dependencies] *solve problems on their own but other parts of the country demand special favours*; (4) *People in* [England/Wales/Scotland/Northern Ireland/Crown Dependencies] *have less say in the future of the country compared to people in other parts of the country*; (5) *The UK Government does not listen to the needs and wishes of the people in* [England/Wales/Scotland/Northern Ireland/Crown Dependencies]; (6) *People in* [England/Wales/Scotland/Northern Ireland/Crown Dependencies] *have a distinct culture that is often misunderstood by people elsewhere in the UK*; (7) *People in the rest of the UK do not understand the culture and lifestyle of people living in* [England/Wales/Scotland/Northern Ireland/Crown Dependencies]; (8) *The way the British system of government concentrates power in Westminster is responsible for causing many problems*; (9) *It would be better if* [England/Wales/Scotland/Northern Ireland/Crown Dependencies] *were an independent country rather than part of the larger United Kingdom.* Responses to all items were scored on a 5-point Likert scale ranging from (1) strongly disagree to (5) strongly agree.

*2.2.9.14 Contemporary UK/Irish political issues.*

*2.2.9.14.1 Border poll - Ireland*. Two items were generated to ask respondents living Northern Ireland (only) about their views on a border poll for the island of Ireland: (1) Would you support a Border Poll to determine whether Northern Ireland should leave the UK and become part of the Republic of Ireland? Responses were scored on a 5-point Likert scale ranging from (1) strongly oppose to (5) strongly support. And (2) If there were a Border Poll to determine whether Northern Ireland should leave the UK and become part of the Republic of Ireland, how would you vote? Responses options were: (i) I would vote for Northern Ireland to remain part of the UK; (ii) I would vote for Northern Ireland to re-join the Republic of Ireland; and (iii) I don’t know how I would vote.

*2.2.9.14.2 Scottish Independence.* Respondents living in Scotland (only) were asked: “*Would you support another Independence Referendum for Scotland*?”, with response options ranging from (1) strongly oppose to (5) strongly support. These respondents were also asked “*How would you vote in this Referendum?* Responses options were: (i) I would vote for Scotland to remain part of the UK; (ii) I would vote for Scotland to be an independent country; and (iii) I don’t know how I would vote.

*2.2.9.14.3 Regions of England*. Respondents living in England (only) were asked: “*Do you consider yourself to be a Northerner, a Southerner, or neither?* Responses options were: (i) I consider myself a Northerner; (ii) I consider myself a Southerner; and (iii) I consider myself neither. Next, these respondents were also asked three questions relating to culture, resources, and future, as follows: (1) *How much do you think the culture in your region of England is misunderstood in other parts of England?* (response options ranged from 1, very misunderstood to 4, not at all misunderstood); (2) *To what extent do you think your region of England gets its fair share of resources compared to other parts of England?* (response options ranged from 1, not at all to 4, to a great extent); and (3) *To what extent do you think the people in your region of England have a say in the future of the country compared to people in other parts of England?* (response options ranged from 1, not at all to 4, to a great extent).

*2.2.9.14.4 View on the Union.* Respondents were asked two questions about the future of the Union, both of which were scored on a visual slider scale ranging from 0, very unlikely to 100, very likely (centred at 50): (1) *Within your lifetime, how likely do you think it is that England, Scotland, Wales, and Northern Ireland will remain one united country?*; and (2) *Within your lifetime, how much do you want England, Scotland, Wales, and Northern Ireland to remain one united country?*

*2.2.9.14.5 Ireland’s response to the pandemic.* Respondents in Northern Ireland (only) were asked “*Do you think that there should be an all-Ireland strategy for handling the pandemic?* (yes/no response option).

*2.2.9.15 European Referendum.* A series of questions were asked to obtain respondents’ views on the European Referendum held in 2016. The first question, adapted from a 2020 YouGov political poll (Smith, 2020), asked respondents to consider: “*In hindsight, do you think Britain was right or wrong to vote to leave the EU?,* with responses scored on a 7-point Likert scale ranging from (1) very wrong to (7) very right. Next, a series of questions were generated to assess respondents their views on the UK’s future after the process of leaving the EU is completed in 2020. Seven statements were presented as follows: *After leaving the EU, do you think (1) the UK’s economic position will be… (2) The UK’s international political standing will be… (3) The UK’s ability to control immigration and the border will be… (4) The UK’s trade deals will be… (5) The UK’s ability to respond effectively to international outbreaks of disease will be… (6) The UK’s ability to respond to security threats will be… and (7) The relationship between the countries in the UK (England, Wales, Scotland, and Northern Ireland) will be…* with respondents’ answers scored using a 5-point Likert scale ranging from 1, a lot worse to 5 much better.

*2.2.9.16 Identity with the European Union*. Respondents were presented with a series of four statements, all scored on a 5-point Likert scale ranging from 1, strongly disagree to 5, strongly agree, as follows: (1) *I identify with people who voted to remain in the EU in the 2016 referendum*; (2) *I feel strong ties with people who voted to remain in the EU in the 2016 referendum*; (3) *I identify with people who voted to leave the EU in the 2016 referendum*; and (4) *I feel strong ties with people who voted to leave the EU in the 2016 referendum*.

*2.2.9.17 End of Brexit Transition period.* Respondents were asked to make two predictions about life after the Brexit Transition period ends on 31 December 2020, as follows: (1) In your opinion, how likely is it that there will be serious disruption, hold ups, and lorry queues at the UK’s borders during January 2020*? and (2) All foods imported from the EU will continue to be imported and available in supermarkets throughout January 2020*? Both statements were scored using a visual slider scale ranging from 0, impossible to 100, certain (centred on 50, chances about even). (*Note. There was a typographical error in the survey in terms of the year, should have been 2021, following the end of the Brexit Transition period on 31 December 2020).

*2.2.10 Trust.* Respondents were asked the extent to which they have trust in the following institutions/groups: (1) UK parliament; (2) the UK government; (3) the devolved government in Wales; (4) the devolved government in Scotland; (5) the devolved government in Northern Ireland; (6) your local government (council or local authority); (7) the police; (8) the legal system; (9) political parties; (10) scientists; (11) doctors and other health professionals and (12) pharmaceutical companies. Responses were scored on a 5-point Likert scale ranging from 1 ‘do not trust at all’ to 5 ‘completely trust’.

*2.2.11 Seasonal items - Christmas holiday period 2020*. The C19PRC-UKW4 fieldwork was conducted approximately one-month prior to the Christmas holiday period 2020, the first such period to happen during the pandemic. A series of questions were derived to assess respondents’ and circumstances and needs in relation to this upcoming festive period. First, respondents presented with two statements: “*Thinking about the upcoming Christmas holiday period and your ability to afford: (1) food* and (separately) (2) *presents, are you*”, (1) Not at all worried to (10) Extremely worried. Respondents were then asked to indicate (by responding on a 5-point Likert scale ranging from 1, not at all difficult to 5, extremely difficulty)resented with two statements: “*Thinking about the upcoming Christmas holiday period and being able to find (1) food to buy; and (2) presents to buy.*

*2.2.12 Survey List Experiment*. At the end of the survey, a survey list experiment was conducted to assess respondents’ engagement or compliance with COVID-19 related public health measures during lockdown. The items were generated by the C19PRC study team using standard survey list experiment techniques for measuring sensitive behaviour (Imai, Unknown; Rosenfeld, Imai, & Shapiro, 2016). The randomisation process occurred as follows: two separate final sections were presented to respondents, List A contained four statements, while List B contained five statements (all of the items from List A and an additional treatment item to assess the sensitive behaviour, indicated in List B by * below). Prior to each List being presented, the following statement was shown: *We would now like to ask you how willing you are to break rules or conventions. Please look at the following list of common rules and indicate how many of these you have done in the last 6 months. LIST: (1) I have driven a car at more than 100 miles an hour; (2) I have travelled illegally to North Korea; (3) I have sometimes not paid my bills on time; (4) I have borrowed something from a friend and forgotten to return it; (5*) I have socialised in another household during lockdown.* Respondents completed the experiment by reporting the number of rules or conventions that they have broken in the last 6 months.”

**References**

Adler, N. E., Epel, E. S., Castellazzo, G., & Ickovics, J. R. (2000). Relationship of subjective and objective social status with psychological and physiological functioning: Preliminary data in healthy, White women. *Health Psychology, 19*(6), 586.

Alsuhibani, A., Shevlin, M., & Bentall, R. P. ((in submission)). Atheism is not the absence of religion: Development of the Monotheist and Atheist Belief Scales and associations with death anxiety and analytic thinking. .

American Psychiatric Association. (2000). *Diagnostic criteria from dsM-iV-tr*: American Psychiatric Pub.

Beck, A. T., Weissman, A., Lester, D., & Trexler, L. (1974). The measurement of pessimism: the hopelessness scale. *Journal of consulting and clinical psychology, 42*(6), 861.

Bell, D. N., & Blanchflower, D. G. (2021). Underemployment in the United States and Europe. *ILR Review, 74*(1), 56-94.

Ben‐Ezra, M., Karatzias, T., Hyland, P., Brewin, C. R., Cloitre, M., Bisson, J. I., . . . Shevlin, M. (2018). Posttraumatic stress disorder (PTSD) and complex PTSD (CPTSD) as per ICD‐11 proposals: A population study in Israel. *Depression and anxiety, 35*(3), 264-274.

Bizumic, B., & Duckitt, J. (2018). Investigating Right Wing Authoritarianism with a Very Short Authoritarianism scale. *Journal of Social and Political Psychology*.

Blanchflower, D. G., & Clark, A. E. (2020). *Children, unhappiness and family finances. GLO Discussion paper, No. 561.* Retrieved from Essen, Germany: <https://cpb-us-e1.wpmucdn.com/sites.dartmouth.edu/dist/5/2216/files/2020/09/GLO-DP-05611.pdf>

British Election Study. (2019). *British Election Study 2014-2024. Combined waves 1-15 internet panel codebook.* Retrieved from: <https://www.britishelectionstudy.com/wp-content/uploads/2019/06/Bes_wave15Documentation_V2.pdf>

British Social Attitudes Survey. (2015). Questionnaire. Retrieved from <http://doc.ukdataservice.ac.uk/doc/8116/mrdoc/pdf/8116_bsa2015_documentation.pdf>

Castanho Silva, B., Jungkunz, S., Helbling, M., & Littvay, L. (2020). An empirical comparison of seven populist attitudes scales. *Political Research Quarterly, 73*(2), 409-424.

Cloitre, M., Hyland, P., Bisson, J. I., Brewin, C. R., Roberts, N., Karatzias, T., & Shevlin, M. (2019). ICD-11 PTSD and complex PTSD in the United States: a population-based study. *Journal of Traumatic Stress*.

Cloitre, M., Shevlin, M., Brewin, C. R., Bisson, J. I., Roberts, N. P., Maercker, A., . . . Hyland, P. (2018). The International Trauma Questionnaire: development of a self‐report measure of ICD‐11 PTSD and complex PTSD. *Acta Psychiatrica Scandinavica, 138*(6), 536-546.

Cohrs, J. C., Maes, J., Moschner, B., & Kielmann, S. (2007). Determinants of human rights attitudes and behavior: A comparison and integration of psychological perspectives. *Political Psychology, 28*(4), 441-469.

Contoyannis, P., Jones, A. M., & Rice, N. (2004). The dynamics of health in the British Household Panel Survey. *Journal of Applied Econometrics, 19*(4), 473-503.

Davidov, E. (2011). Nationalism and constructive patriotism: A longitudinal test of comparability in 22 countries with the ISSP. *International Journal of Public Opinion Research, 23*(1), 88-103.

Davis, M. H. (1980). *Interpersonal reactivity index*: Edwin Mellen Press.

Davis, M. H. (1983). Measuring individual differences in empathy: Evidence for a multidimensional approach. *Journal of personality and social psychology, 44*(1), 113.

Duckitt, J. (2001). A dual-process cognitive-motivational theory of ideology and prejudice. In *Advances in experimental social psychology* (Vol. 33, pp. 41-113): Elsevier.

Duckitt, J. (2009). Authoritarianism and dogmatism. *Handbook of individual differences in social behavior, 298317*.

Elahi, A., Algorta, G. P., Varese, F., McIntyre, J., & Bentall, R. (2017). Do paranoid delusions exist on a continuum with subclinical paranoia? A multi-method taxometric study. *Schizophrenia Research, 190*, 77-81.

European Social Survey. (2015). Round 7 Module on attitudes towards immigration and their antecdents - question design final module in template. Retrieved from <https://www.europeansocialsurvey.org/docs/round7/questionnaire/ESS7_immigration_final_module_template.pdf>

Everson, S. A., Goldberg, D. E., Kaplan, G. A., Cohen, R. D., Pukkala, E., Tuomilehto, J., & Salonen, J. T. (1996). Hopelessness and risk of mortality and incidence of myocardial infarction and cancer. *Psychosomatic medicine, 58*(2), 113-121.

Fieldhouse, E., Green, J., Evans, G., Schmitt, H., van der Eijk, C., Mellon, J., & Prosser, C. (2016). British Election Study, 2015: Face-to-Face Post-Election Survey.

Fieldhouse, E., Green, J., Evans, G., Schmitt, H., van der Eijk, C., Mellon, J., & Prosser, C. (2018). *British Election Study, 2017: Face-to-Face Post-Election Survey* Retrieved from <https://www.britishelectionstudy.com/wp-content/uploads/2019/01/BES-2017-F2F-codebook.pdf>

Fraser, L., Burnell, M., Salter, L. C., Fourkala, E.-O., Kalsi, J., Ryan, A., . . . Menon, U. (2014). Identifying hopelessness in population research: a validation study of two brief measures of hopelessness. *BMJ open, 4*(5).

Frederick, S. (2005). Cognitive reflection and decision making. *Journal of Economic perspectives, 19*(4), 25-42.

Ho, A. K., Sidanius, J., Kteily, N., Sheehy-Skeffington, J., Pratto, F., Henkel, K. E., . . . Stewart, A. L. (2015). The nature of social dominance orientation: Theorizing and measuring preferences for intergroup inequality using the new SDO₇ scale. *Journal of Personality and Social Psychology, 109*(6), 1003.

Huddy, L., & Khatib, N. (2007). American patriotism, national identity, and political involvement. *American journal of political science, 51*(1), 63-77.

Hughes, M. E., Waite, L. J., Hawkley, L. C., & Cacioppo, J. T. (2004). A short scale for measuring loneliness in large surveys: Results from two population-based studies. *Research on aging, 26*(6), 655-672.

Hyland, P., Shevlin, M., Brewin, C. R., Cloitre, M., Downes, A., Jumbe, S., . . . Roberts, N. (2017). Validation of post‐traumatic stress disorder (PTSD) and complex PTSD using the International Trauma Questionnaire. *Acta Psychiatrica Scandinavica, 136*(3), 313-322.

Imai, K. (Unknown). Examples of List Experiments. Retrieved from <https://imai.fas.harvard.edu/research/files/listExamples.pdf>

Imhoff, R., & Bruder, M. (2014). Speaking (un‐) truth to power: Conspiracy mentality as a generalised political attitude. *European Journal of Personality, 28*(1), 25-43.

Kahneman, D. (2012). Thinking fast and slow (UK edition). In: London: Penguin.

Karatzias, T., Shevlin, M., Fyvie, C., Hyland, P., Efthymiadou, E., Wilson, D., . . . Cloitre, M. (2016). An initial psychometric assessment of an ICD-11 based measure of PTSD and complex PTSD (ICD-TQ): Evidence of construct validity. *Journal of Anxiety Disorders, 44*, 73-79.

Kossowska, M., Trejtowicz, M., de Lemus, S., Bukowski, M., Van Hiel, A., & Goodwin, R. (2011). Relationships between right‐wing authoritarianism, terrorism threat, and attitudes towards restrictions of civil rights: A comparison among four European countries. *British Journal of Psychology, 102*(2), 245-259.

Kroenke, K., Spitzer, R. L., & Williams, J. B. (2001). The PHQ‐9: validity of a brief depression severity measure. *Journal of general internal medicine, 16*(9), 606-613.

Kroenke, K., Spitzer, R. L., Williams, J. B., & Löwe, B. (2010). The patient health questionnaire somatic, anxiety, and depressive symptom scales: a systematic review. *General hospital psychiatry, 32*(4), 345-359.

Löwe, B., Decker, O., Müller, S., Brähler, E., Schellberg, D., Herzog, W., & Herzberg, P. Y. (2008). Validation and standardization of the Generalized Anxiety Disorder Screener (GAD-7) in the general population. *Medical care*, 266-274.

McIntyre, J. C., Wickham, S., Barr, B., & Bentall, R. P. (2018). Social identity and psychosis: Associations and psychological mechanisms. *Schizophrenia bulletin, 44*(3), 681-690.

McManus, S., Bebbington, P., Jenkins, R., & Brugha, T. (2016). *Mental health and wellbeing in England: Adult psychiatric morbidity survey 2014.* Retrieved from Leeds:

Melo, S., Corcoran, R., Shryane, N., & Bentall, R. P. (2009). The persecution and deservedness scale. *Psychology and Psychotherapy: Theory, Research and Practice, 82*(3), 247-260.

Menasce Horowitz, J. (2019). The American Trends Panel Survey Methodology. *Social & Demographic Trends.* Retrieved from <https://www.pewresearch.org/social-trends/2019/05/08/methodology-20/>

Munis, B. K. (2020). Us Over Here Versus Them Over There… Literally: Measuring Place Resentment in American Politics. *Political Behavior*, 1-22. doi:10.1007/s11109-020-09641-2

Office for National Statistics. (2016). Personal well-being in the UK: 2015 to 2016. Personal well-being findings from the Annual Population Survey (APS), with analysis by country, region and individual characteristics. . Retrieved from <https://www.ons.gov.uk/peoplepopulationandcommunity/wellbeing/bulletins/measuringnationalwellbeing/2015to2016>

Parker, C. S. (2010). Symbolic versus blind patriotism: Distinction without difference? *Political Research Quarterly, 63*(1), 97-114.

Pulos, S., Elison, J., & Lennon, R. (2004). The hierarchical structure of the Interpersonal Reactivity Index. *Social behavior and personality, 32*(4), 355-360.

Radloff, L. S. (1977). The CES-D scale: A self-report depression scale for research in the general population. *Applied psychological measurement, 1*(3), 385-401.

Rammstedt, B., & John, O. P. (2007). Measuring personality in one minute or less: A 10-item short version of the Big Five Inventory in English and German. *Journal of research in Personality, 41*(1), 203-212.

Rosenfeld, B., Imai, K., & Shapiro, J. N. (2016). An empirical validation study of popular survey methodologies for sensitive questions. *American journal of political science, 60*(3), 783-802.

Schatz, R. T. (1994). On being a good American: Blind versus constructive patriotism.

Seeman, T. E., Miller-Martinez, D. M., Stein Merkin, S., Lachman, M. E., Tun, P. A., & Karlamangla, A. S. (2011). Histories of social engagement and adult cognition: midlife in the US study. *Journals of Gerontology Series B: Psychological Sciences and Social Sciences, 66B*(suppl_1), i141-i152. doi:doi.org/10.1093/geronb/gbq091

Sirota, M., & Juanchich, M. (2018). Effect of response format on cognitive reflection: Validating a two-and four-option multiple choice question version of the Cognitive Reflection Test. *Behavior research methods, 50*(6), 2511-2522.

Smith, F. (2020). With the transition period almost over, by 51% to 40% Britons think we were wrong to vote to leave. Retrieved from <https://yougov.co.uk/topics/politics/articles-reports/2020/12/22/transition-period-almost-over-51-40-britons-think->

Spitzer, R. L., Kroenke, K., Williams, J. B., & Löwe, B. (2006). A brief measure for assessing generalized anxiety disorder: the GAD-7. *Archives of internal medicine, 166*(10), 1092-1097.

Stewart-Brown, S., Tennant, A., Tennant, R., Platt, S., Parkinson, J., & Weich, S. (2009). Internal construct validity of the Warwick-Edinburgh mental well-being scale (WEMWBS): a Rasch analysis using data from the Scottish health education population survey. *Health and quality of life outcomes, 7*(1), 1-8.

UK Cabinet Office/Department of Education. (2020). Guidance: Critical workers who can access schools or educational settings. Retrieved from <https://www.gov.uk/government/publications/coronavirus-covid-19-maintaining-educational-provision/guidance-for-schools-colleges-and-local-authorities-on-maintaining-educational-provision>

UK Government Coronavirus (COVID-19) in the UK. (2021). Vaccinations in the United Kingdom. Retrieved from <https://coronavirus.data.gov.uk/details/vaccinations>

US Census Bureau. (2020). Source of the data and accuracy of the estimates for the 2020 Household Pulse Survey - Phase 2. Interagency Federal Statistical Rapid Response Survey to Measure Household Experiences during the Coronavirus (COVID-19) pandemic. . Retrieved from <https://www2.census.gov/programs-surveys/demo/technical-documentation/hhp/Phase2_Source_and_Accuracy_Week_17.pdf>

Vallières, F., Ceannt, R., Daccache, F., Abou Daher, R., Sleiman, J., Gilmore, B., . . . Hyland, P. (2018). ICD‐11 PTSD and complex PTSD amongst Syrian refugees in Lebanon: the factor structure and the clinical utility of the International Trauma Questionnaire. *Acta Psychiatrica Scandinavica, 138*(6), 547-557.
