# Supplementary material for: Design, content, and fieldwork procedures of the COVID‐19 Psychological Research Consortium (C19PRC) Study – Wave 4
Source: Int J Methods Psychiatr Res. 2021 Nov 5;31(1):e1899. doi: 10.1002/mpr.1899 (PMC8646695; doi:10.1002/mpr.1899)
Supplement: Supplementary file 2 — Supporting Information S2 [file MPR-31-e1899-s002.docx]

Design, content, and fieldwork procedures of the COVID-19 Psychological Research Consortium (C19PRC) Study – Wave 4

Orla McBride^1*^, Sarah Butter^2^, Jamie Murphy^1^, Mark Shevlin^1^, Todd K. Hartman^2^, Kate M. Bennett^3^, Thomas VA Stocks^2^, Alex Lloyd^4^, Ryan McKay^4^, Jilly Gibson-Miller^2^, Liat Levita^2^, Liam Mason^5^, Anton P. Martinez^2^, Philip Hyland^6^, Frédérique Vallières^7^, Thanos Karatzias^8^, Carmen Valiente^9^, Carmelo Vazquez^9^, & Richard P. Bentall^2^

**Supplementary Results and Tables**

^1^ Ulster University, Northern Ireland

^2^ University of Sheffield, England

^3^ University of Liverpool, England

^4^ Royal Holloway, University of London, England

^5^ University College London, England

^6^ Maynooth University, Republic of Ireland

^7^Trinity College Dublin, Republic of Ireland

^8^Napier University, Scotland

^9^Complutense University of Madrid

Short title: C19PRC Study Wave 4 protocol

**Corresponding author:** Dr Orla McBride, School of Psychology, Ulster University, Cromore Road, Coleraine, BT52 1SA, Northern Ireland. Email: [o.mcbride@ulster.ac.uk](mailto:o.mcbride@ulster.ac.uk); Tel: 0044(0)2870123987.

**3. Results**

Supplementary Table 1 displays the outcome of the raking weighting procedure for the C19PRC-UKW4 data. The counts and percentages for baseline sampling quotas determined for gender, age, and household income, as well as the proportions of baseline respondents according to urbanicity, ethnicity, household composition and born/raised in the UK are presented in the first column. The counts and percentages of baseline respondents recontacted at C19PRC-UKW4 (fourth wave) are presented in the middle column. The weighted counts and frequencies having applied the weight variable generated from the raking procedure (see Main Text, section **2.4 Data analysis plan and weight procedures.**) is presented in the final column. Applying the weight variable for the longitudinal panel strand (N=1271) at C19PRC-UKW4 successful rebalances the sample to within 1% (see Main Text, section **3.2 Weight procedures Phase 1 longitudinal panel from baseline).**

Supplementary Table 1. Outcome of raking weighting procedure at C19PRC-UKW4

| C19PRC-UKW1 (baseline) sociodemographic characteristics | C19PRC-UKW1 (*N=*2025) | C1PRC-UKW1 respondents recontacted at C19PRC-UKW4 (not weighted; *N*=1271 | C1PRC-UKW1 respondents recontacted at C19PRC-UKW4 (weighted – 5% weight; *N*=1271) |
| --- | --- | --- | --- |
| **Gender** |  |  |  |
| Male | 972 (48.0%) | 635 (50.0%) | 601 (47.3%) |
| Female | 1047 (51.7%) | 635 (50.0%) | 670 (52.7%) |
| Other | 6 (0.3%) | 1 (0.1%) | - |
| **Age (years)** |  |  |  |
| 18-24 | 246 (12.1%) | 77 (6.1%) | 154 (12.1%) |
| 25-34 | 380 (18.8%) | 191 (15.0%) | 239 (18.8%) |
| 35-44 | 353 (17.4%) | 210 (16.5%) | 222 (17.4%) |
| 45-54 | 410 (20.2%) | 294 (23.1%) | 257 (20.2%) |
| 55-64 | 349 (17.2%) | 268 (21.1%) | 219 (17.2%) |
| 65+ | 287 (14.2%) | 231 (18.2%) | 180 (14.2%) |
| **2019 Annual income** |  |  |  |
| £0-£15490 | 410 (20.2%) | 264 (20.8%) | 258 (20.3%) |
| £15,491-£25,340 | 410 (20.2%) | 235 (18.5%) | 257 (20.2%) |
| £25,341-£38,740 | 385 (19.0%) | 237 (18.6%) | 241 (18.9%) |
| £38,741-£57,930 | 410 (20.2%) | 271 (21.3%) | 262 (20.6%) |
| £57,931+ | 410 (20.2%) | 264 (20.8%) | 254 (20.0%) |
| **Urbanicity** |  |  |  |
| City | 498 (24.6%) | 268 (21.1%) | 313 (24.6%) |
| Suburb/Town/Rural | 1527 (75.4%) | 1003 (78.9%) | 958 (75.4%) |
| **Ethnicity** |  |  |  |
| White | 1848 (91.2%) | 1186 (93.3%) | 1170 (92.0%) |
| Non-white | 177 (8.7%) | 85 (6.7%) | 101 (8.0%) |
| **Household composition** |  |  |  |
| Children in household | 592 (29.2%) | 329 (25.9%) | 372 (29.2%) |
| No children in household | 1433 (70.8%) | 942 (74.1%) | 899 (70.8%) |
| **Born or raised in UK** |  |  |  |
| Yes | 1891 (93.4%) | 1201 (94.5%) | 1198 (94.2%) |
| No | 134 (6.6%) | 70 (5.5%) | 73 (5.8%) |

C19PRC-UKW4 Phase 2 sampling quotas were set to target females, younger adults, and lower income earners for the ‘top-up’ sample. As presented in Supplementary Table 2, this process was successful - combining the Phase 1 sample with ‘top-up’ respondents from Phase 2 produced a cross-sectional sample which closely mirrored the distribution of the UK adult population in terms of gender (to within 0.7-0.9%, more females in the sample), age (to within 0.3-2.3%, fewer adults aged 18-24 years in the sample), and household income (to within 0.2-1.5%, with more lower income earners in the sample).

Supplementary Table 2 Outcome of quota sampling recruitment, COVID-19 Psychological Research Consortium (C19PRC) Study UK Wave 4 (C19PRC-UKW4), November – December 2020

| Socio-demographic characteristics used for quota sampling | | Quotas  C19PRC-UKW1 | C19PRC-UKW4 Phase 1 (*N*=1796) | | C19PRC-UKW4 Phase 2 ‘top-up’ (*N*=292) | | C19PRC-UKW4  Sample  (Phases 1 & Phase 2 ‘top-up’)  (*N*=2088) | | Percentage difference between quota target and quota obtained at C19PRC-UKW4 |
| --- | --- | --- | --- | --- | --- | --- | --- | --- | --- |
|  |  | % | n | % | n | % | n | % |  |
| Sex† | Men | 49 | 877 | 48.8 | 127 | 43.5 | 1004 | 48.1 | -0.9 |
|  | Women | 51 | 919 | 51.2 | 160 | 54.8 | 1079 | 51.7 | +0.7 |
|  | Other | NA | 0 | 0.0 | 5 | 1.7 | 5 | 0.2 | NA |
| Age group (years) † | 18-24 | 12 | 114 | 6.3 | 88 | 30.1 | 202 | 9.7 | -2.3 |
|  | 25-34 | 19 | 314 | 17.5 | 98 | 33.6 | 412 | 19.7 | +0.7 |
|  | 35-44 | 18 | 339 | 18.9 | 30 | 10.3 | 369 | 17.7 | -0.3 |
|  | 45-54 | 20 | 394 | 21.9 | 30 | 10.3 | 424 | 20.3 | +0.3 |
|  | 55-64 | 17 | 347 | 19.3 | 28 | 9.6 | 375 | 18.0 | +1.0 |
|  | 65+ | 14 | 288 | 16.0 | 18 | 6.2 | 306 | 14.7 | +0.7 |
| Gross annual household income ‡ | £0-£15490 | 20 | 388 | 21.6 | 56 | 19.2 | 444 | 21.3 | +1.3 |
|  | £15,491-£25,340 | 20 | 323 | 18.0 | 80 | 27.4 | 403 | 19.3 | -0.7 |
|  | £25,341-£38,740 | 20 | 343 | 19.1 | 79 | 27.1 | 422 | 20.2 | +0.2 |
|  | £38,741-£57,930 | 20 | 394 | 21.9 | 39 | 13.4 | 433 | 20.7 | +0.7 |
|  | £57,931+ | 20 | 348 | 19.4 | 38 | 13.0 | 386 | 18.5 | -1.5 |

†Quotas for age and sex were derived from EUROSTAT 2016 population estimates (Eurostat, 2020)

‡Quotas for gross household income bands were on 2016 Office for National Statistics data (Office for National Statistics, 2017)

The socio-demographic characteristics and voting behaviours of new respondents recruited as part of the Phase 2 oversample are presented in Supplementary Table 3. This process successfully recruited a diverse sample of adults from across the three smaller UK nations to facilitate meaningful between-country socio-political analyses. To demonstrate the comparability of the sample composition to population estimates, we use Scotland as an example because England and Wales population estimates are typically combined in national data sources, and comparable data for some indicators are not publicly available for Northern Ireland. When we compared the Scotland strand of the Phase 2 oversample to most recently available population estimates, the sample closely matched the corresponding population characteristics in terms of: gender (female: 51.5% sample vs. 51.8% of adult population), age (65+ years; 27.1% in sample vs. 23% of adult population), nationality (born outside UK: 7.3% sample vs. 8% in population), and household composition (single adult household: 25.3% in sample vs. 33.1% in population) (National Records Scotland, 2017, 2020, 2021). In addition, for all three nations (Scotland, Wales, and Northern Ireland), we identified the political party which obtained the highest proportion of respondents’ first preference votes in the 2019 General Election, by country, in the Phase 2 oversample and compared this data to the final election result. The results closely mirrored the final election result in that, in the oversample, the Conservative Party in Wales, the Scottish National Party in Scotland, and the Democratic Unionist Party in Northern Ireland, had the highest proportion of first preference votes (UK Parliament House of Commons Library, 2019). Finally, we compared the 2016 EU Referendum results by country and found that, by and large, data obtained from the Phase 2 oversample closely mirrored the final EU Referendum outcome (BBC, 2016): the large majority vote to remain in the EU compared to leave the EU following the 2016 EU Referendum was evident for Northern Ireland (45.1% remain vs. 33.7% leave) and Scotland (55.6% remain vs 30.2% leave), whereas for Wales, although there was a majority vote to remain, this only exceeded the vote to leave by 0.5% (44.2% leave vs. 43.7% remain).

Supplementary Table 3 Socio-demographic and political voting characteristics of respondents recruited in Phase 2 ‘booster’ strand in C19PRC-UKW4 (N=1779)

| Respondent characteristics | | **C19PRC-UKW4 Booster Strand (N=1779)** | | |
| --- | --- | --- | --- | --- |
|  |  | **Wales (N=588)** | **Scotland (N=586)** | **Northern Ireland (N=605)** |
|  |  | **N(%)** | **N(%)** | **N(%)** |
| Gender | Male | 286 (48.6%) | 279 (47.6%) | 291 (48.1%) |
|  | Female | 300 (51.0%) | 302 (51.5%) | 310 (51.2%) |
|  | Other | 2 (0.4%) | 5 (0.9%) | 4 (0.7%) |
| Age (years) | 18-24 | 27 (4.6%) | 38 (6.5%) | 63 (10.4%) |
|  | 25-34 | 56 (9.5%) | 73 (12.5%) | 93 (15.4%) |
|  | 35-44 | 86 (14.6%) | 97 (16.6%) | 100 (16.5%) |
|  | 45-54 | 105 (17.9%) | 94 (16.0%) | 97 (16.0%) |
|  | 55-64 | 157 (26.7%) | 125 (21.3%) | 154 (25.5%) |
|  | 65+ | 157 (26.7%) | 159 (27.1%) | 98 (16.2%) |
| 2019 annual  household income | ≤£15.490 | 132 (22.4%) | 132 (22.4%) | 135 (22.3%) |
|  | £15,491-£25,340 | 149 (25.3%) | 110 (18.8%) | 161 (26.6%) |
|  | £25,341-£38,740 | 131 (22.3%) | 172 (29.4%) | 147 (24.3%) |
|  | £38,741-£57,903 | 114 (19.4%) | 115 (19.6%) | 111 (18.3%) |
|  | ≥£57,931 | 62 (10.5%) | 57 (9.7%) | 51 (8.4%) |
| Ethnicity | White | 575 (97.8%) | 567 (96.8%) | 587 (97.0%) |
|  | Non-white | 13 (2.2%) | 19 (3.2%) | 18 (3.0%) |
| Born in UK | Yes | 570 (96.9%) | 543 (92.7%) | 570 (94.2%) |
|  | No | 18 (3.1%) | 43 (7.3%) | 35 (5.8%) |
| Household composition | Living alone | 130 (22.1%) | 148 (25.3%) | 144 (23.8%) |
|  | Not living alone | 458 (77.9%) | 438 (74.7%) | 461 (76.2%) |
| 2016 EU referendum vote | Voted to leave the EU | 257 (43.7%) | 177 (30.2%) | 204 (33.7%) |
|  | Voted to stay in the EU | 260 (44.2%) | 326 (55.6%) | 273 (45.1%) |
|  | Did not vote | 57 (9.7%) | 49 (8.4%) | 103 (17.0%) |
|  | Ineligible (too young) | 8 (1.4%) | 18 (3.1%) | 16 (2.6%) |
|  | Ineligible (not UK citizen/resident) | 6 91.0%) | 16 (2.7%) | 9 (1.5%) |
| Brexit hindsight vote | Very/somewhat/slightly wrong | 281 (47.8%) | 345 (58.9%) | 339 (56.0%) |
|  | Neither right nor wrong | 71 (12.1%) | 61 (10.4%) | 67 (11.1%) |
|  | Very/somewhat/slightly right | 236 (40.1%) | 180 (30.7%) | 199 (32.9%) |
| General election 2019 vote | Ineligible to vote | 12 (2.0%) | 29 (4.9%) | 24 (4.0%) |
|  | Eligible to vote but did not vote | 57 (9.7%) | 48 (8.2%) | 88 (14.5%) |
|  | Alliance Party of Northern Ireland | 1 (0.2%) | - | 100 (16.5%) |
|  | BREXIT Party | 22 (3.7%) | 10 (1.7%) | 5 (0.8%) |
|  | Conservative and Unionist Party | 205 (34.9%) | 130 (22.2%) | 20 (3.3%) |
|  | Democratic Unionist Party | 1 (0.2%) | 1 (0.1%) | 121 (20.0%) |
|  | Green Party | 11 (1.9%) | 14 (2.4%) | 23 (3.8%) |
|  | Labour Party | 186 (31.6%) | 84 (14.3%) | 11 (1.8%) |
|  | Plaid Cymru | 65 (11.1%) | - | - |
|  | Scottish National Party | - | 214 (36.5%) | 1 (0.2%) |
|  | Sinn Féin | - | - | 65 (10.7%) |
|  | Social Democratic and Labour Party | - | 9 (1.5%) | 53 (8.8%) |
|  | UKIP | - | 1 (0.2%) | - |
|  | Ulster Unionist Party | - | 1 (0.2%) | 64 (10.6%) |
|  | Other | 28 (4.8%) | 45 (7.7%) | 30 (5.0%) |
| Political party most identify with | Alliance Party of Northern Ireland | 1 (0.2%) | - | 92 (15.2%) |
|  | BREXIT Party | 17 (2.9%) | 8 (1.4%) | 6 (1.0%) |
|  | Conservative and Unionist Party | 170 (28.9%) | 110 (18.8%) | 31 (5.1%) |
|  | Democratic Unionist Party | 2 (0.3%) | 3 (2.8%) | 101 (16.7%) |
|  | Green Party | 27 (4.6%) | 25 (4.3%) | 27 (4.5%) |
|  | Labour Party | 193 (32.8%) | 89 (15.2%) | 18 (3.0%) |
|  | Plaid Cymru | 69 (11.7%) | - | - |
|  | Scottish National Party | - | 232 (39.6%) | 5 (0.8%) |
|  | Sinn Féin | - | - | 57 (9.4%) |
|  | Social Democratic and Labour Party | 2 (0.3%) | 3 (0.5%) | 52 (8.6%) |
|  | UK Independence Party (UKIP) | 3 (0.5%) | 3 (0.5%) | 1 (0.2%) |
|  | Ulster Unionist Party | - | 1 (0.2%) | 63 (10.4%) |
|  | Other | 17 (2.9%) | 33 (5.6%) | 22 (3.6%) |
|  | None | 87 (14.8%) | 79 (13.5%) | 130 (21.5%) |

References

BBC. (2016). EU Referendum Results. Retrieved from <https://www.bbc.co.uk/news/politics/eu_referendum/results>

Eurostat. (2020). Population on 1 January by age and sex. Retrieved from <https://appsso.eurostat.ec.europa.eu/nui/show.do?dataset=demo_pjan&lang=en>

National Records Scotland. (2017). Scotland’s Population. The Registrar General’s Annual Review of Demographic Trends. Retrieved from <https://www.nrscotland.gov.uk/files/statistics/rgar/16/16rgar.pdf>

National Records Scotland. (2020). Mid-population estimates Scotland. Retrieved from <https://www.nrscotland.gov.uk/statistics-and-data/statistics/statistics-by-theme/population/population-estimates/mid-year-population-estimates/mid-2019>

National Records Scotland. (2021). *Population by Country of Birth and Nationality, Scotland, July 2019 to June 2020* Retrieved from Edinburgh, Scotland.: <https://www.nrscotland.gov.uk/files//statistics/population-estimates/pop-cob-nat-19-20/pop-cob-nat-19-20-report.pdf>

Office for National Statistics. (2017). Household disposable income and inequality in the UK: financial year ending 2016. Retrieved from <https://www.ons.gov.uk/peoplepopulationandcommunity/personalandhouseholdfinances/incomeandwealth/bulletins/householddisposableincomeandinequality/financialyearending2016>

UK Parliament House of Commons Library. (2019). Insight: General Election 2019: The results. Retrieved from <https://commonslibrary.parliament.uk/general-election-2019-the-results-so-far/>
